# First Attempt to Couple Proteomics with the *AhR* Reporter Gene Bioassay in Soil Pollution Monitoring and Assessment

**DOI:** 10.3390/toxics10010009

**Published:** 2021-12-29

**Authors:** Claudia Landi, Giulia Liberatori, Pietro Cotugno, Lucrezia Sturba, Maria Luisa Vannuccini, Federica Massari, Daniela Valeria Miniero, Angelo Tursi, Enxhi Shaba, Peter A. Behnisch, Alfonso Carleo, Fabrizio Di Giuseppe, Stefania Angelucci, Luca Bini, Ilaria Corsi

**Affiliations:** 1 Department of Life Sciences, University of Siena, 53100 Siena, Italy; landi35@unisi.it (C.L.); enxhi.shaba@unisi.it (E.S.); 2 Department of Physical, Earth and Environmental Sciences, University of Siena, 53100 Siena, Italy; giulia.liberatori@student.unisi.it (G.L.); sturba@student.unisi.it (L.S.); marialuisa.vannuccini@unisi.it (M.L.V.); 3 Department of Biology, University of Bari Aldo Moro, 70121 Bari, Italy; pietro.cotugno@uniba.it (P.C.); federica.massari@uniba.it (F.M.); danielavaleria.miniero@uniba.it (D.V.M.); angelo.tursi@uniba.it (A.T.); 4 BioDetection System BV (BDS) Amsterdam, 1098 XH Amsterdam, The Netherlands; Peter.Behnisch@bds.nl; 5 Department of Pulmonology, Hannover Medical School, 30625 Hannover, Germany; alfonsocarleo@yahoo.it; 6 Department of Medical, Oral & Biotechnological Sciences, Dentistry and Biotechnology and Proteomics Unit, Centre of Advanced Studies and Technology, “G. D’Annunzio”, University of Chieti-Pescara, 66100 Chieti, Italy; f.digiuseppe@unich.it (F.D.G.); s.angelucci@unich.it (S.A.)

**Keywords:** bioassay, proteomics, polyhalogenated compounds, contaminants of emerging concern, soil pollution, risk assessment

## Abstract

A topsoil sample obtained from a highly industrialized area (Taranto, Italy) was tested on the DR-CALUX^®^ cell line and the exposed cells processed with proteomic and bioinformatics analyses. The presence of polyhalogenated compounds in the topsoil extracts was confirmed by GC-MS/MS analysis. Proteomic analysis of the cells exposed to the topsoil extracts identified 43 differential proteins. Enrichment analysis highlighted biological processes, such as the cellular response to a chemical stimulus, stress, and inorganic substances; regulation of translation; regulation of apoptotic process; and the response to organonitrogen compounds in light of particular drugs and compounds, extrapolated by bioinformatics all linked to the identified protein modifications. Our results confirm and reflect the complex epidemiological situation occurring among Taranto inhabitants and underline the need to further investigate the presence and sources of inferred chemicals in soils. The combination of bioassays and proteomics reveals a more complex scenario of chemicals able to affect cellular pathways and leading to toxicities rather than those identified by only bioassays and related chemical analysis. This combined approach turns out to be a promising tool for soil risk assessment and deserves further investigation and developments for soil monitoring and risk assessment.

## 1. Introduction

Chemically activated luciferase gene expression (CALUX^®^) in vitro cell bioassays represent suitable bioanalytical tools in screening and relative quantification of legacy and emerging contaminants in environmental matrices and biological samples [1,2,3]. In particular, the Dioxin Responsive (DR-CALUX^®^) bioassay has been successfully used to detect polyhalogenated compounds [4,5] and widely applied to screen human and environmental matrices [4,6,7,8]. However, there is still room for a further implementation of such reliable effect-based tools, since rat hepatoma cells (H4IIE) are responsive to other chemicals and/or their complex mixtures present in sample extracts and activate molecular and biochemical responses by different cellular pathways [9,10]. Both the European Food Safety Authority (EFSA) and the European Chemical Agency (ECHA) recently recognized the importance of including effect-based tools in monitoring and hazard assessment of chemical mixtures as those mostly present in environmental matrices [11]. Moreover, current drawbacks in the analytical detection of contaminants of emerging concerns (CECs) and threshold effects, which are almost unknown, strongly encourage the application of bioassays as more sensitive tools for a wide range of chemical compounds [12]. Chemicals present in the sample extract other than those detected by the reporter gene are still able to activate cellular pathways and/or interfere with those of dioxin and dioxin-like compounds; therefore, they could still be identified. Given this, proteomics can help in this regard, and through bioinformatics, it can link the proteomic profile to selected chemicals known for affecting specific cellular pathways [13,14,15,16,17]. Environmental risk assessment of chemical mixtures is even more challenging [18], and current approaches are still limited to single-chemical toxicities [19] rather than of mixtures [19,20]. Furthermore, in assessing risk, analytical chemistry cannot identify synergism/antagonism among physical and chemical stressors; therefore, effect-based tools are far better descriptors of environmental exposure scenarios [21,22]. Being widely recommended in environmental risk assessment [23,24,25,26], bioassays have been recently proposed as a quantitative measure of mixture effects’ toxicity for environmental samples, such as soil, wastewaters, and wastes [21,27,28,29,30]. Here, we aim to use proteomics coupled with the DR-CALUX^®^ bioassay to monitor the presence of contaminants in the topsoil and the risks for human and environmental health. The proteomics approach allows us to extrapolate differentially abundant proteins regulated upon exposure to a contaminated topsoil sample rather than only using the bioanalytical toxicity equivalents (BEQs) obtained by the DR-CALUX^®^ system itself. Furthermore, the soil extract was further processed by GC-MS/MS to detect legacy pollutants according to the Italian National Regulation as PCDD/Fs; PCBs, including dioxin-like PCBs; polycyclic aromatic hydrocarbons (PAHs); pesticides; and C > 12 hydrocarbons (Italian National Laws D.M. 46/2019 and D.Lgs. 152/2006).

## 2. Materials and Methods

### 2.1. Chemicals

All cell reagents were purchased from Gibco-Life Technologies (Carlsbad, CA, USA). Solvents used for the extraction and purification of soil samples were pesticide residue grade and obtained from Sigma-Aldrich (St. Louis, MO, USA) and Biosolve Chimie (Dieuze, France). Labeled standards for chemical analysis were purchased from Cambridge Isotope Laboratories (Andover, MA, USA) and Cerilliant (Round Rock, TX, USA). The 2,3,7,8-tetrachlorodibenzo-*p*-dioxin (TCDD) standard used for the bioassay calibration curve was acquired from Wellington Laboratories (Guelph, ON, Canada). Reagents for 2DE and mass spectrometry analysis were obtained from Sigma-Aldrich (St. Louis, MO, USA), GE Healthcare (Uppsala, Sweden), and Bio-Rad Laboratories (Hercules, CA, USA).

### 2.2. Experimental Design

Two topsoil samples (20 cm depth) were collected in 2018 in a 20 m^2^ area in proximity to the largest industrial steel and iron ore sinter plant of Taranto (Apulia Region, South Italy) and to illegal dumping sites and the steel plant’s landfills nearby. Located in the south-east of Italy, the city of Taranto has been included among the National Priority Contaminated Sites and as an area of high risk of environmental crisis (defined within Italian National Laws D.Lgs. n. 426/98; D.M. 10 January 2000). A composite topsoil sample (5 kg) made up of 3 sub-samples taken in a 20 m^2^ area was obtained using a stainless-steel spade. The resulting topsoil composite sample (60 g) was lyophilized, sieved (2 mm), and extracted (5 g) by accelerated solvent extraction (Dionex ASE 350, Thermo Fisher Scientific) using a standardized protocol previously described [31]. One topsoil extract was tested using the traditional luciferase measurement conducted by following the Dutch standard method RIKZ-SPECIE 07 according to the method reported for soil samples in [31]. The other topsoil extract, after the same procedure of extraction and purification, was used in proteomic analysis.

### 2.3. Rat Hepatoma Cell (H4IIE) Preparation for Proteomic Analysis

For proteomic analysis, rat hepatoma cells (H4IIE), transfected with the *AhR*-controlled luciferase reporter gene construct (pGudLuc1.1; DR-CALUX^®^), were exposed in a 6-well plate to each topsoil extract (DxCS), respectively, one for PCDD/Fs and one for *dl*-PCBs. This modification in the soil standard methods was made in order to obtain enough amount of protein for quantification. A control sample with only cells in the medium was included (CTRL) as well as a sample made with the addition of 0.8% DMSO, which is generally used as a carrier for dioxin and dioxin-like extracts. For each sample, 3 replicates were run. After 24 h, exposure media were transferred to a falcon tube, cells were washed, trypsin was added, and cells were resuspended in the growth medium. Falcon tubes were centrifuged for 5 min at 1100× *g* at room temperature and this step repeated 3 times with 1 mL of PBS using the same protocol. PBS was then discarded, and pellets were stored at −80 °C until use. Cells pellets were resuspended for proteomic analysis in 70 μL of lysis buffer (7M urea, 2M thiourea, 4% (*w*/*v*) CHAPS, and 1% (*w*/*v*) DTE) to extract the whole protein content. Protein concentration was then estimated using Bradford assay [31] before proceeding with two-dimensional electrophoresis. All sample aliquots were stored at −80 °C until use.

### 2.4. High-Resolution 2D Electrophoresis

Two-dimensional electrophoresis on pH 3–10 nonlinear, immobilized pH gradient strips and image analyses were carried out, as reported by [17]. 2DE was carried out using the Immobiline polyacrylamide system on a preformed immobilized nonlinear pH gradient, from pH 3 to 10, 18 cm in length, from GE Healthcare (Uppsala, Sweden). Samples for analytical runs were loaded by rehydration loading and analyzed using the Ettan™ IPGphor™ system (Amersham Biosciences) at 16 °C under the following electrical conditions: 30 V for 8 h, 200 V for 1 h, a gradient until 3500 V for 2 h, and a step of 3500 V for other 2 h. After that, a gradient was applied until 5000 V for 2 h and maintained at 5000 V for other 3 h, another gradient until 8000 V for 1 h, and a step of 8000 V for 3 h. In the end, analysis was performed at a gradient until 10,000 V for 1 h and maintained for a total of 90,000 Vh. Preparative strips were rehydrated with 350 μL of lysis buffer, and sample loading was performed adding 2% *v*/*v* carrier ampholytes by cup-loading with the cup applied at the cathodic ends of the strips. After the first dimensional run, the IPG gels were equilibrated in 6M urea, 2% *w*/*v* SDS, 2% *w*/*v* DTE, 30% *v*/*v* glycerol, and 0.05 M Tris-HCl at pH 6.8 for 12 min and for a further 5 min in 6 M urea, 2% *w*/*v* SDS, 2.5% *w*/*v* iodoacetamide, 30% *v*/*v* glycerol, 0.05 M Tris-HCl at pH 6.8, and a trace of bromophenol blue. Second-dimensional separation was performed on 9–16% polyacrylamide linear gradient gels (18 × 20 cm × 1.5 mm) and carried out at a 40 mA/gel constant current at 9 °C until the dye front reached the bottom of the gel. Analytical gels were stained with ammoniacal silver nitrate. MS-preparatory gels were stained with MS-compatible silver staining. Gels were then digitized with an Image Scanner III laser densitometer controlled by LabScan 6.0 software (GE Healthcare, Uppsala, Sweden). Computer-aided 2D image analysis was carried out with the Image Master Platinum 6.0 computer system (GE Healthcare, Uppsala, Sweden). The analysis process was performed by matching all gels of each group with a reference gel for the same condition, having the best resolution and the highest number of spots, chosen by the user and named “master” by the software. The master reference gels were then matched with each other to perform the inter-class analysis. By this procedure, the Image Master Platinum algorithm matched all the gels to find quantitative and qualitative differences.

Spots were considered differentially abundant when the percentage of relative volume (%V) means ratio was greater than 1.8-fold with a valid statistical test by non-parametric Kruskal–Wallis analysis (*p* ≤ 0.05), followed by Dunn’s test showing also *p*-adjusted and z-value (RStudio Desktop 1.1.463; Integrated Development for RStudio, Inc., Boston, MA, USA, https://www.rstudio.com, accessed on 27 January 2021).

### 2.5. Mass Spectrometry by MALDI ToF-ToF

Detected spot differences were excised from MS-compatible silver staining gel and destained in 2.5 mM ammonium bicarbonate and 50% acetonitrile (ACN) and dehydrated in acetonitrile. Subsequent protein spots were digested in 50 mM NH_4_HCO_3_ containing trypsin and incubated overnight at 37 °C. Peptide extract was applied to a C18ZipTip (Millipore, CA, USA), rinsed with a 0.1% TFA, and eluted directly on the MALDI target with 0.5 μL of a saturated α-cyano-4-hydroxycinnamic acid (1:1 = ACN: 0.1% TFA) solution. Tryptic digests were analyzed by an Autoflex™ Speed mass spectrometer (Bruker Daltonics, Bremen, Germany), as earlier reported by [32], equipped with a Nd:YAG laser (355 nm, 1000 Hz) operated by FlexControl v3.3 and equipped with a 355 nm nitrogen laser. All spectra were obtained with the delayed extraction technology in positive reflectron mode and averaged from 100 laser shots to improve the S/N ratio. External high-precision calibration was performed using a peptide mixture containing bradykinin (fragments 1–7) 757.39 *m*/*z*, angiotensin II 1046.54 *m*/*z*, ACTH (fragments 18–39) 2465.19 *m*/*z*, Glu fibrinopeptide B 1571.57 *m*/*z*, and renin substrate tetradecapeptide porcine 1760.02 *m*/*z*. Samples analyzed by PMF were additionally analyzed using LIFT MS/MS from the same target. Analyses were performed in positive LIFT reflectron mode. The precursor ion selector range was 0.65% of the parent ion mass. The voltage parameters were set at IS1 6 kV, IS2 5.3 kV, lens 3.00 kV, reflector 1 27.0 kV, reflector 2 11.45 kV, LIFT1 19 kV, and LIFT 2 4.40 kV. Following MS acquisition, each spectrum was submitted to PMF for protein database searches through the Mascot search engine (Matrix Science Ltd., London, UK, http://www.matrixscience.com, accessed on 18 March 2021) online software using combined PMF and MS/MS datasets via BioTools 3.2 (Bruker Daltonics). Mascot compares the experimentally determined tryptic peptide masses with theoretical peptide masses calculated for protein from these databases. Search parameters were as follows: Swiss-Prot/TrEMBL and NCBInr as databases; taxonomy limited to *Rattus* or *Mus musculus*; a peptide mass fingerprint enzyme, trypsin; fixed modification, carbamidomethylation (Cys); variable modifications, oxidation of methionine; mass values, monoisotopic; ion charge state set to +1; maximum miscleavages set to 1; and mass tolerance of 100 ppm for PMF and 0.6–0.8 Da for MS/MS. The mass spectrometry proteomics data have been deposited to the ProteomeXchange Consortium via the PRIDE partner repository with the dataset identifier PXD027074.

### 2.6. PCA and Heatmap Analysis

Differential spots were used to perform multivariate analysis by principal component analysis (PCA). To visualize the behavior of the differentially abundant spots in each gel of the considered conditions, heatmap analysis was performed using the %V values of the statistically significant differentially abundant spots. In particular, the clustering of protein spots was performed using Euclidean distance. The above-mentioned analyses and the related figures were obtained by RStudio Desktop 1.1.463 (Integrated Development for RStudio, Inc., Boston, MA, USA, https://www.rstudio.com, accessed on 16 February 2021).

### 2.7. Enrichment Analyses

#### 2.7.1. Gene Ontology Terms by DAVID

The list of the accession numbers of the identified proteins underwent functional analysis by the DAVID Bioinformatic Resources (6.8) online tool (https://david.ncifcrf.gov/, accessed on 6 April 2021) to understand the biological meaning behind the large list of proteins. A functional annotation tool was chosen, and our accession numbers were uploaded selecting “uniprot_accession” as the identifier and “gene list” as the list type. Gene Ontology was selected among the Annotation Summary Results.

#### 2.7.2. Enrichr

Enrichr software is a comprehensive resource for curated gene sets, freely available at http://amp.pharm.mssm.edu/Enrichr, accessed on 20 April 2021 [33]. Search engines accumulated biological knowledge for further biological discoveries, thanks to 339,127 terms, 171 libraries, and 32,220,066 lists analyzed. To perform the analysis, we submitted the gene names of the identified proteins. Enrichr contains many features and datasets, such as Transcription, Pathways, Ontologies, Diseases/Drugs, and Cell Types. In particular, for our analysis, we took into consideration results obtained by Pathways and Disease/Drug datasets. From Pathways was selected BioPlanet2019, and from Disease/Drug was chosen DSigDB. Both report statistical data by *p*-value, *p*-adjusted, and related genes that refer to the specified class.

#### 2.7.3. UniProt BLAST for Human Proteins Similarity

To find human proteins with higher similarity with the identified rat and mouse proteins, we performed a BLAST analysis using the UniProt tool. Once the accession number of the identified protein was submitted on UniProtKB (https://www.uniprot.org/, accessed on 3 May 2021), we performed BLAST, selecting blastp as the program, blosum62 as the matrix, and 10 as the threshold. The database of reference was SwissProt.

#### 2.7.4. Disease (by Biomarkers) Analysis by MetaCore

Identified protein spots and similar human proteins were further processed by the MetaCore 6.8 network building tool (Clarivate Analytics) that includes a manually annotated database of protein interactions, metabolic reactions, and diseases obtained from the scientific literature. The accession numbers of the two lists of proteins were uploaded into MetaCore and processed. Enrichment analysis was based on the hypergeometric distribution algorithm, and relevant “process networks” and “diseases (by biomarkers)” results were then prioritized according to their statistical significance and compared among the two lists of proteins.

### 2.8. Chemical Analysis of Topsoil Samples

The analytical determinations of PCDDs, PCDFs, and *dl*-PCBs were carried out according to EPA methods 1613B and 1668C, using a Trace™ 1300 gas chromatograph (Thermo Fisher Scientific) coupled to mass spectrometry (TSQ 8000, Triple Quadrupole, Thermo Fisher Scientific) according to the methods already fully described in [31] for soil samples. Estimated concentrations for each detected analyte of PCDD/Fs and *dl*-PCBs were expressed in terms of toxic equivalency (TEQ), resulting from the product of the concentration and individual toxic equivalent factor (TEF) of each congener [34]. For PCBs, the sum of the selected congener concentrations was also reported as the total amount of PCBs, expressed as μg/kg dry weight (d.w.). Detailed information for the extraction, clean-up, and quantitation methods of soil samples for GC-MS/MS analysis of PCDD/Fs and *dl*-PCBs are reported in the Supplementary Material. Polycyclic aromatic hydrocarbons (PAHs) were also determined according to what is required by the Italian National Regulation, D.Lgs. 152/2006, according to the 8270 EPA method. Details on extraction, clean-up analytical procedures, and instrumental analysis by GC-MS/MS are reported in the Supplementary Material (Tables S5 and S6). The determination and quantitation of C > 12 hydrocarbons was performed according to procedure no. 75/2011 reported by the Institute for Environmental Protection and Research (ISPRA, available at accessed on 1 October 2021). The extraction and clean-up procedures and details of the GC-FID analytical method are reported in the Supplementary Material.

## 3. Results

### 3.1. DR-CALUX^®^ Bioassay and GC-MS/MS Analysis of Topsoil Extracts

The presence of PCDD/Fs and *dl*-PCBs in the topsoil extracts was confirmed by both BEQ values obtained from DR-CALUX^®^ bioassay and TEQ_WHO_ from GC-MS/MS. Mean BEQ values were 10.6 ± 2.2 ng 2,3,7,8-TCDD BEQ/kg d.w., and the sum of TEQ_WHO_ values was 59 ± 17 ng TEQ/kg d.w., with furans being the main contributors (70%), followed by dioxins (23.3%) (Table 1). The presence of other carcinogenic and mutagenic compounds, such as PAHs and C > 12 hydrocarbons, was also found in the topsoil sample, and ∑PAHs (0.017 mg/kg d.w.) showed the only contribution of benzo[b]fluoranthene (69.4%) and benzo[k]fluoranthene (30.6%) (Table 2), while ∑C > 12 resulted in over 70.4 mg/kg. On the contrary, pesticides resulted in all data below the quantification limits (LOQs) (data not shown).

### 3.2. Proteomics Analysis

**Figure 1:**
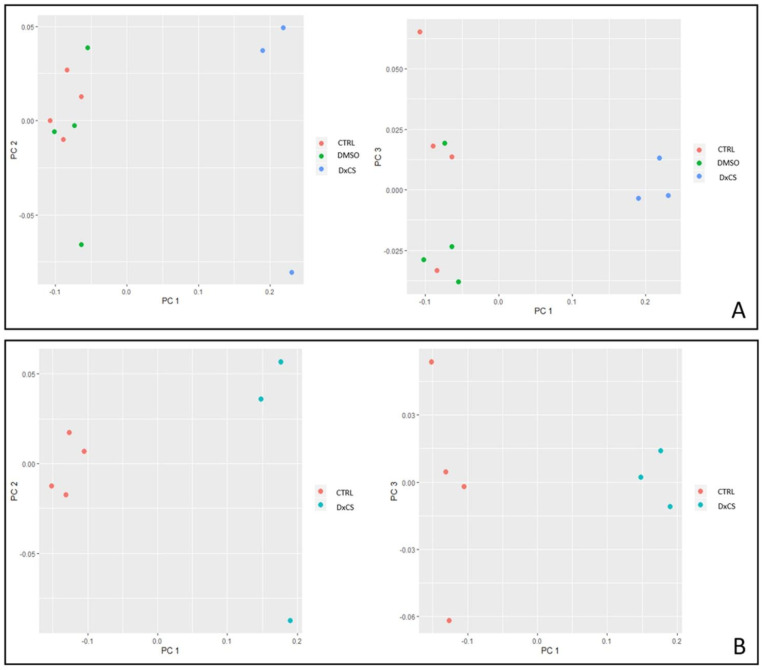
PCA performed with the %V of the differentially abundant spots. (**A**) PCA considering the analyzed 3 conditions. (**B**) PCA considering CTRL vs. DxCS. Both analyses showed 2 different points of view: PC1 and PC2, and PC1 and PC3.

**Figure 2:**
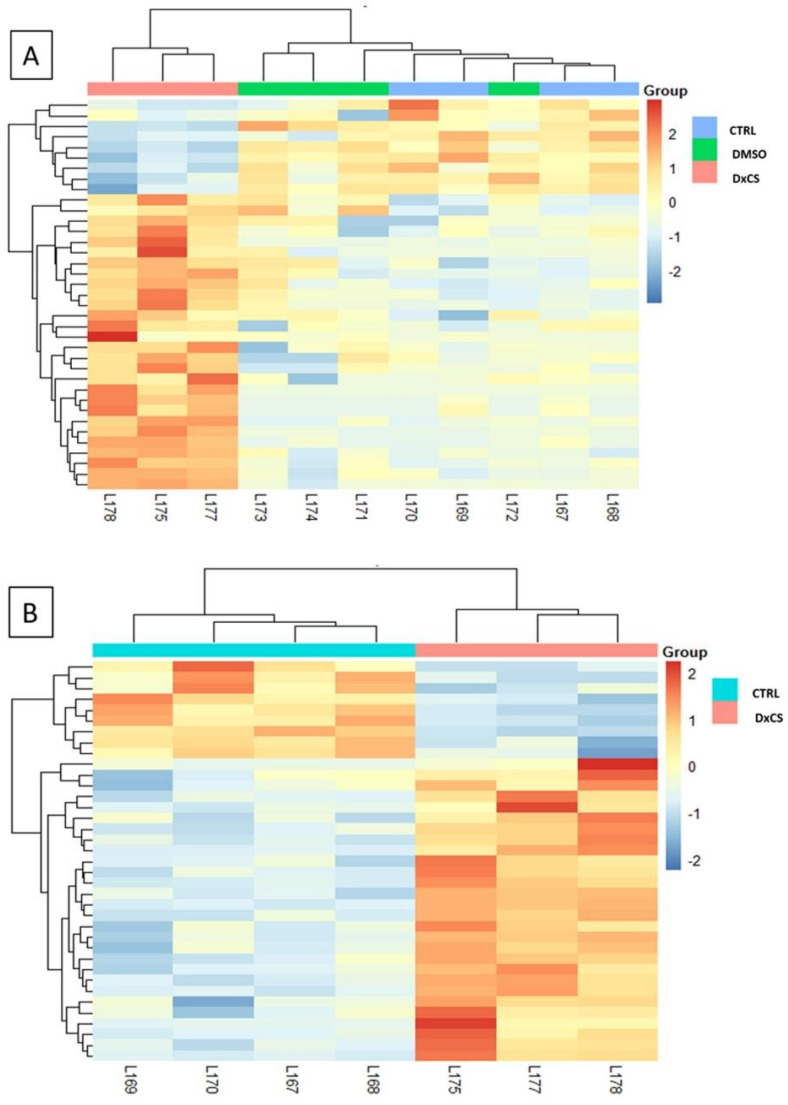
(**A**) Heatmap analysis among the 3 analyzed groups (CTRL, DMSO, DxCS) and (**B**) between CTRL and DxCS.

Proteomic analysis revealed 43 statistically different abundant spots in cells exposed to extracts DxCS (PCDD/Fs and *dl*-PCBs) compared to CTRL (only cells) and DMSO only (0.8) (see Figure S1 for gels and Table S8 for spots analysis). No effects on protein abundance were observed for DMSO vs. CTRL, also confirmed by PCA, while differences were observed between DxCS vs. CTRL and DxCS vs. DMSO (Figure 1A). Figure 1B confirms that CTRL remained quite distinct from DxCS along PC1. In addition, we also performed heatmap analysis (Figure 2). Spot abundance between CTRL and DMSO had a similar distribution when compared with DxCS treatment (Figure 2A). Furtherly, Figure 2B confirmed the opposite trend between CTRL and DxCS.

Protein identification is shown in Table S9, and functional annotation of the Gene Ontology terms of our differential proteins performed by DAVID is shown in Table 3 and summarized as follows: (i) cellular response to chemical stimulus, (ii) cellular response to stress, (iii) response to inorganic substances, (iv) regulation of translation, (v) regulation of apoptotic process, and (vi) response to organonitrogen compounds as the most represented Biological Processes (BP). Moreover, differential proteins came from extracellular exosomes, vesicles, and also from cytoplasmic portions, as reported by “Cellular Components” (CC) results. “Molecular Functions” (MF) analysis reported RNA binding, purine ribonucleoside triphosphate binding, adenyl nucleotide binding, ATP binding, and nucleotide binding.

Further, we performed enrichment analysis by Enrichr-highlighting chemical substances by DSigDB and molecular pathways by the BioPlanet2019 database related to the differential proteins. Results are reported in Table 4 and Table 5, respectively.

**Figure 3:**
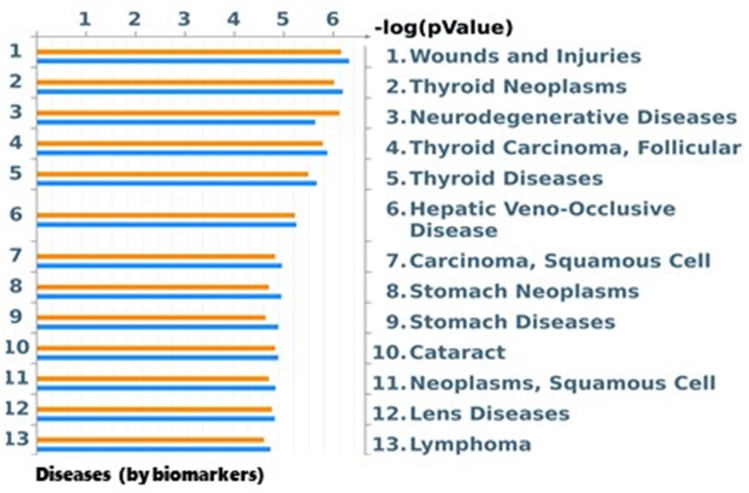
Enrichment analysis by MetaCore of Diseases (by Biomarkers). Orange histograms represent enrichment for human homolog proteins. Blue histograms represent enrichment for differential rat proteins.

Because some contaminants, such as dioxins, have important human health effects, we also performed a protein BLAST of the rat and mouse differential proteins in order to find homologous human proteins reported in Table S9. With the two lists of proteins (rat and human), we performed a new enrichment analysis using MetaCore software in order to extrapolate both “Disease (by Biomarkers)” results. Figure 3 reports the histograms representing the probability that our proteins are involved in a particular pathology. Orange and blue histograms refer, respectively, to human and rat protein lists. As shown, histograms were similar, reporting a generic wound and injuries, but also thyroid neoplasms, neurodegenerative diseases, and other types of cancers, such as squamous cell carcinoma, stomach neoplasm, and lymphoma.

## 4. Discussion

The application of high-throughput effect-based tools, such as in vitro bioassays, could help to assess ecological and human risks associated with the exposure to legacy contaminants and CECs, and they will be thus useful as screening tools for monitoring complex environmental matrices as more relevant pollution scenarios [21]. To this aim, we proposed a combined approach of proteomics coupled with DR-CALUX^®^ bioassay in a topsoil sample collected from the Taranto industrial area. Taranto is well known from the past as one of the most productive historical districts in Italy. It includes the largest integrated steelworks in Europe, which is, according to [36], the main factor responsible for Italy’s total emission of PCDD/Fs and *dl*-PCBs. In the same areas, a crude oil refinery, three power plants, the third largest naval port of Italy, and a cement factory are also located. Such anthropic activities not only have been estimated to produce approx. 3.25 Mt of solid waste and by-products [37], but more recently, illegal landfills [38] of building materials containing asbestos, accidental oil spills and burning [39], and the storage of hydrocarbons and hazardous wastes have been also reported [40,41].

The DR-CALUX^®^ BEQ values obtained from this study confirm such sources of contamination, since they are comparable to those reported for contaminated soils in Taiwan [35] and similar to those found in open-burning surface soils in Vietnam [35]. GC-MS/MS analysis confirmed bioassay responses and showed that levels of ∑PCDD/F/*dl*-PCBs are above Italian National Regulatory Limits set at 6 ng TEQ/kg d.w. in agricultural and farming soils (D.M. 46/2019), as well as those of ∑PCDD/Fs set at 10 ng TEQ/kg d.w. in green, private, and recreational used soils (Italian National Law D.Lgs. 152/2006). Carcinogenic PAHs were also found, such as benzo[b]fluoranthene and benzo[k]fluoranthene, which show the highest REP (relative potencies) among all PAHs [42]. Moreover, C > 12 hydrocarbons also showed above regulatory limits set as 50 mg/kg according to the Italian National Law (D.M. 46/2019 and D.Lgs. 152/2006).

Concerning results of the applied proteomics, once proved that 0.8% of DMSO does not alter the differential protein pattern compared to CTRL and that 43 proteins showed altered abundance in cells exposed to topsoil extracts (DxCS), enrichment analysis was performed on these proteins. It allowed us to identify a series of biological processes associated with exposure to particular chemicals or compounds able to alter molecular functions related to nucleic acid and ATP binding and associated with diseases, mostly cancers. Most of the deregulated proteins seem to be present on extracellular organelles, such as exosomes (EEs) and vesicles (EVs), which play a key role in cell-to-cell communication and are reported to be enhanced upon exposure to environmental toxins or carcinogens [43]. According to the enrichment analysis, the biological processes that we found could be affected by tanespimicin, a drug used in younger patients with different types of leukemia [44], and thapsigargin, specifically used against prostate cancer. Similarly, lomustine, an active compound used to treat tumors during chemotherapy, seems to be involved, as well as fluorouracil, one of the most used anticancer chemotherapy agent in cancer clinics in the adjuvant therapy of pancreatic tumors, the latter highly documented in the Taranto male population [39]. Other suggested chemicals, such as vorinostat used for the treatment of cutaneous T cell non-Hodgkin lymphoma and combined with other drugs for brain tumors, can be inferred [45]. Moreover, the analysis allowed us to identify troglitazone and glibenclamid, respectively, anti-diabetic and anti-inflammatory drugs; cyclosporine to block the rejection of organ transplantation; and clonidine, lobeline, and chlorpromazine all used in the treatment of drug dependence [46,47,48]. The presence of potassium dichromate as well as pesticides was also inferred. The first one is probably linked to its use in several industrial settings both in steel plants and in the military sector. The presence of pesticides was detected by GC-MS/MS analysis, though the results were below quantification limits. However, enrichment analysis also suggested the presence of the herbicide atrazine, forbidden in European countries since 1992 but probably still present in soil due to illegal disposal or past contamination being also persistent in the environment [49]. Although the soil extracts suitable for luciferase measurement in DR-CALUX^®^ bioassay are obtained with a mixture of highly hydrophobic and hydrophilic solvents during extraction and clean-up operations to isolate the fraction of dioxin-like chemicals, we cannot exclude that some drugs could have been retained and/or that the identified pathways obtained by the proteomics and enrichments analysis could be still activated by other dioxin-like compounds present in the extracts. In fact, based on the Enrichr analysis, the most inferred chemicals are hydrophobic as their molecular lipophilicity (log P) result > 0 (Table S7), having found compounds with log P ranging from 1.04 of clindamycin to 5.5 of troglitazone.

Moreover, proteomic results reveal a deterioration of cellular proteostasis significantly associated with tumorigenesis [50] suggested by the up-regulation after DxCS exposure of IF4E, HYOU1, HS105, TRAP1, SYAC, and UBA1 and down-regulation of ASNS and EF2. Protein folding, translocation, and degradation, as well as the assembly and disassembly of protein complexes, were principally promoted by molecular chaperones, such as HS105 and TRAP1, whose up-regulation could indicate the presence of ER stress as well as the alternate energy metabolism in cancer cells [51]. Interestingly, their increased gene expression levels were observed in rat and human lung epithelial cells after Cr (VI) exposure [52], suggesting that potassium dichromate, whose exposure was inferred from the enrichment analysis, is involved in proteostasis alteration. Moreover, HYOU1 belongs to a family implicated in the heat shock protein (HSP) cellular response to environmental stress and involved in protein folding, with a pivotal role in cytoprotective cellular mechanisms triggered by oxygen deprivation [53] and regulating the secretion of vascular endothelial growth factor (VEGF) to drive the progression of angiogenesis [53]. Being reported to be significantly up-regulated in different cancers [54], it is also induced by rosiglitazone [55], an anti-diabetic drug, a derivative of troglitazone, that we found. Enrichment analysis also suggests a response to hypoxia and oxidative stress by HYOU1, MVP, GSTM2, quinone oxidoreductase-like protein 1, TST, BLVRB, and PRDX1 up-regulation in cells exposed to soil extracts (DxCS), as well as BLVRB related to heme degradation [56,57,58]. Interestingly, atrazine could be the cause of the observed oxidative stress [49].

Most of the differential proteins identified refer to signal transduction processes. In particular STRAP, TRAP1, and PAK2 refer to TGF-β signaling [59,60], whose alteration contributes to many diseases, including cancer and fibrosis [16,61]. The results obtained from proteomic and enrichment analyses of soil extracts showed specific cellular pathways linked to diseases that are mostly documented in the Taranto inhabitants [35,39,62,63]. Further studies should be then conducted in Taranto topsoil, with the aim to detect the presence of drugs and chemicals recognized by Enrich analysis to confirm their presence and more importantly their sources. Mismanagement of wastes (e.g., illegal dumping sites), including those deriving from the city hospital of Taranto, have been recently documented in the Taranto territory, and it cannot be excluded that some of them will end up in the soil [64]. In our previous study, by applying DR-CALUX^®^ in Taranto topsoil samples [30], we documented the presence of *dl*-PCBs, probably originating from illegal dumping operations of Apirolio^®^, a PCBs-based cooling liquid mostly containing *dl*-PCBs as well as asbestos resins and furans originating from industrial and other local sources.

As a further analysis, we investigated which pathologies could arise from the differential protein pattern observed in rat hepatoma cells (H4IIE) exposed to soil extracts. Results indicated wounds and injuries as the most significant, thus suggesting the link with repairing mechanisms. Furthermore, several other diseases were recognized, such as thyroid neoplasm, neurodegenerative diseases, hepatic veno-occlusive diseases, squamous cell carcinoma, stomach neoplasm, cataract, lens disease, and lymphomas, all supporting the hypothesis that the differential protein pattern observed is led by carcinogenic processes. In particular, some of them, such as thyroid neoplasm, squamous cell carcinoma, stomach neoplasm, and lymphomas, are also associated with exposure to dioxins and dioxin-like compounds [65]. In the past 20 years, Taranto inhabitants have been the subject of many epidemiological studies that have emphasized the close relationship between environmental pollution and the high incidence of human pathologies [36,38,62,63]. Lung and pleural cancers have been highly documented and identified in industrial plant workers and in those inhabitants living closest to the iron and steel plant and associated carcinogenic pollutant emission sites [66,67,68,69,70]. The high incidence of cancer is also documented in the young population and also in children [38,62,63]. Prospective life and the perception of living in an unhealthy environment may have contributed over the years to the use of anti-depressant and anti-psychotic drugs among Taranto inhabitants [71], such as imipramine, desipramin, fluoxetine, and chlorprothixen.

## 5. Conclusions

The proposed innovative approach of coupling proteomics with an *AhR*-based bioassay in a topsoil sample from a polluted area allowed us to not only detect the presence of PCDD/F_S_, dioxin-like compounds, and PAHs, confirmed by GC-MS/MS analysis, but also to identify several pathways affected by a number of chemicals, including selected pesticides, which resulted below detection limits by GC-MS/MS and pharmaceuticals, which showed a clear link with the epidemiological data of Taranto inhabitants. Results from proteomic and enrichment analyses in fact confirm the complex epidemiological situation of diseases occurring among Taranto inhabitants and underline the need to further investigate their presence and sources in Taranto soils. The documented mismanagement of hospital wastes in some illegal dumping areas of the municipality territory could be a potential source of such contaminations. Coupling effect-based tools, such as DR-CALUX^®^, with proteomics could represent a suitable new tool for risk assessment analysis of complex matrices, such as soils, also for monitoring purposes. Further validation campaigns are thus recommended to reinforce the suitability of the proposed combined approach to be used to assess the occurrence of complex mixtures, including drugs, and their impact on living beings, including humans, depending on the use of soil.

## Figures and Tables

**Table 1:** Total concentrations of PCDDs, PCDFs, and *mono-ortho* and *non-ortho dl*-PCBs expressed as ng/kg d.w., toxic equivalents expressed as TEQ_WHO_ of 2,3,7,8-TCDD as reported in [34], and pattern profile expressed as percentage (%), calculated from GC-MS/MS analysis.

Compound	Concentrations (ng/kg d.w.)	TEQ_WHO_	Percentage (%)
Chlorinated dibenzo-p-dioxins			
2,3,7,8-TCDD	3.5	3.5	5.9%
1,2,3,7,8-PeCDD	9.2	9.2	15.5%
1,2,3,4,7,8-HxCDD	4.4	0.44	0.7%
1,2,3,7,8,9-HxCDD	4.8	0.48	0.8%
1,2,3,4,6,7,8-HpCDD	25.7	0.257	0.4%
1,2,3,4,6,7,8,9-OCDD	40.2	0.01206	0%
Chlorinated dibenzofurans			
2,3,7,8-TCDF	13.9	1.39	2.3%
1,2,3,7,8-PeCDF	18.9	0.567	1%
2,3,4,7,8-PeCDF	83.4	25.02	42.2%
1,2,3,4,7,8-HxCDF	50.4	5.04	8.5%
1,2,3,6,7,8-HxCDF	31.6	3.16	5.3%
1,2,3,7,8,9-HxCDF	44	4.4	7.4%
2,3,4,6,7,8-HxDF	4.6	0.46	0.8%
1,2,3,4,6,7,8-HpCDF	138.1	1.381	2.3%
1,2,3,4,7,8,9-HpCDF	5.5	0.055	0.1%
1,2,3,4,6,7,8,9-OCDF	219	0.0657	0.1%
Non-ortho–substituted PCBs			
3,3’,4,4’-tetraCB (PCB-77)	16	0	0%
3,4,4’,5-tetraCB (PCB-81)	7	0	0%
3,3’,4,4’,5-pentaCB (PCB-126)	37	3.7	6.2%
3,3’,4,4’,5,5’-hexaCB (PCB-169)	5	0.15	0.3%
Mono-ortho–substituted PCBs			
2,3,3’,4,4’-pentaCB (PCB 105)	47	0	0%
2,3,4,4’,5-pentaCB (PCB 114)	7	0	0%
2,3’,4,4’,5-pentaCB (PCB 118)	67	0	0%
2’,3,4,4’,5-pentaCB (PCB 123)	18	0	0%
2,3,3’,4,4’,5-hexaCB (PCB 156)	45	0	0%
2,3,3’,4,4’,5’-hexaCB (PCB 157)	10	0	0%
2,3’,4,4’,5,5’-hexaCB (PCB 167)	44	0	0%
2,3,3’,4,4’,5,5’-heptaCB (PCB 189)	11	0	0%
**average 59**			
**std. dev. 17**			

**Table 2:** Concentrations of single PAHs and their sum expressed as mg/Kg d.w. in topsoil samples, EC_50_ values based on DR-CALUX^®^, toxic equivalents expressed as reported in [35] as TEQ and percentage.

Compound	Concentrations (mg/kg d.w.)	EC_50_ DR CALUX^®^	TEQ	Percentage (%)
Polycyclic Aromatic Hydrocarbons				
Pirene	0.003			0%
Benzo[a]antracene		0.00011		0%
Crisene	0.004			0%
Benzo[b]fluorantene	0.004	0.00092	0.00000368	69.4%
Benzo[k]fluorantene	0.003	0.00054	0.00000162	30.6%
Benzo[a]pirene		0.00025	-	0%
Benzo[g,h,i]perilene	0.003			0%
Dibenzo[a,h]antracene		0.0011		0%
Indeno[123-c,d]antracene	-	0.00076		0%
Dibenzo[a,e]perilene		-		0%
Dibenzo[a,i]perilene		-		0%
Dibenzo[a,l]perilene		-		0%
Dibenzo[a,h]perilene		-		0%
∑PAHs	0.017	0.0000053		

**Table 3:** Functional annotation by DAVID of the Gene Ontology terms of differential proteins, reporting Biological Processes (BP), Cellular Components (CC), and Molecular Functions (MF). Terms refer to a specific Gene Ontology vocabulary describing Biological Processes, Molecular Functions, and Cellular Components, while % represents the percentage of proteins referring to that particular term with statistical significance (*p*-value) adjusted by the Benjamini test.

Biological Processes Terms	%	*p*-Value	Benjamini
cellular response to chemical stimulus	46.2	1.8 × 10^−5^	1.8 × 10^−2^
cellular response to stress	38.5	3.1 × 10^−5^	1.5 × 10^−2^
response to inorganic substance	26.9	5.7 × 10^−5^	1.9 × 10^−2^
response to oxygen-containing compound	38.5	6 × 10^−5^	1.5 × 10^−2^
response to chemical	57.7	1.2 × 10^−4^	2.4 × 10^−2^
response to stress	46.2	2.8 × 10^−4^	4.5 × 10^−2^
response to endogenous stimulus	34.6	3.9 × 10^−4^	5.3 × 10^−2^
regulation of translation	19.2	4 × 10^−4^	4.7 × 10^−2^
regulation of apoptotic process	30.8	5.2 × 10^−4^	5.6 × 10^−2^
regulation of cellular amide metabolic process	19.2	5.2 × 10^−4^	5 × 10^−2^
regulation of programmed cell death	30.8	5.6 × 10^−4^	4.9 × 10^−2^
posttranscriptional regulation of gene expression	19.2	7.5 × 10^−4^	6 × 10^−2^
regulation of cell death	30.8	9.4 × 10^−4^	6.9 × 10^−2^
organonitrogen compound metabolic process	34.6	9.8 × 10^−4^	6.7 × 10^−2^
**Cellular Component Terms**			
extracellular exosome	60	8.2 × 10^−9^	1.4 × 10^−7^
extracellular vesicle	60	8.8 × 10^−9^	7.6 × 10^−7^
extracellular organelle	60	9 × 10^−9^	5.2 × 10^−7^
membrane-bounded vesicle	64	1.2 × 10^−8^	5 × 10^−7^
vesicle	64	2.3 × 10^−8^	8 × 10^−7^
cytoplasmic part	76	2.4 × 10^−7^	6.9 × 10^−6^
cytosol	48	5.7 × 10^−7^	1.4 × 10^−5^
extracellular region part	60	6.8 × 10^−7^	1.5 × 10^−5^
extracellular region	60	2.6 × 10^−6^	4.9 × 10^−5^
cytoplasm	80	5.3 × 10^−6^	9.1 × 10^−5^
intracellular part	80	7 × 10^−4^	1.1 × 10^−2^
intracellular organelle	76	7.4 × 10^−4^	1.1 × 10^−2^
**Molecular Function Terms**			
RNA binding	36	2.8 × 10^−4^	5 × 10^−2^
purine ribonucleoside triphosphate binding	36	3.9 × 10^−4^	3.5 × 10^−2^
purine ribonucleoside binding	36	4 × 10^−4^	2.4 × 10^−2^
ribonucleoside binding	36	4.1 × 10^−4^	1.9 × 10^−2^
purine nucleoside binding	36	4.1 × 10^−4^	1.9 × 10^−2^
nucleoside binding	36	4.2 × 10^−4^	1.5 × 10^−2^
purine ribonucleotide binding	36	4.6 × 10^−4^	1.4 × 10^−2^
purine nucleotide binding	36	4.8 × 10^−4^	1.3 × 10^−2^
ribonucleotide binding	36	4.9 × 10^−4^	1.1 × 10^−2^
ATP binding	32	6 × 10^−4^	1.2 × 10^−2^
adenyl ribonucleotide binding	32	7.1 × 10^−4^	1.3 × 10^−2^
adenyl nucleotide binding	32	7.4 × 10^−4^	1.2 × 10^−2^
small molecule binding	40	8.6 × 10^−4^	1.3 × 10^−2^

**Table 4:** Enrichment analysis of the identified proteins by Enrichr software. The table reports chemical substances related to our proteins by DSigDB, with relative *p*-values and gene names of differential proteins related to the terms.

Term	*p*-Value	Genes
(17S)-17-hydroxy-13,17-dimethyl-1,2,6,7,8,14,15,16-octahydrocyclopenta[a]phenanthren-3-one CTD 00007088	1.39 × 10^−7^	PDXK; PRDX1; ASNS; TALDO1; HYOU1; EEF2; PAK2; ACTG1
67526-95-8 CTD 00007263	1.83 × 10^−10^	TRAP1; TST; MVP; G3BP1; ANXA5; ASNS; HYOU1; PFAS; ACTG1
Vorinostat CTD 00003560	1.43 × 10^−11^	TRAP1; PRDX1; ANXA5; TALDO1; STRAP; BLVRB
troglitazone CTD 00002415	1.53 × 10^−12^	HSPH1; MVP; PRDX1; ANXA5; ASNS; ACTG1
chlortetracycline HL60 DOWN	2.94 × 10^−12^	PDXK; HSPH1; TST; PRDX1; CRYZL1; UBA1; PAK2
clonidine HL60 DOWN	3.74 × 10^−11^	HSPH1; PFAS; EIF4E
lobeline HL60 DOWN	4.72 × 10^−12^	PDXK; HSPH1; G3BP1; CRYZL1; TALDO1; PFAS; PAK2; EIF4E
PERHEXILINE CTD 00006493	4.79 × 10^−11^	ASNS; EIF4E
POTASSIUM DICHROMATE CTD 00006598	6.61 × 10^−11^	TRAP1; MVP; PRDX1; ASNS; UBA1
atrazine CTD 00005450	6.7 × 10^−11^	TRAP1; GSTM2; HSPH1; TST; MVP; PRDX1; G3BP1; ANXA5; CRYZL1; ASNS; PFAS
cyproheptadine PC3 UP	7.38 × 10^−11^	ASNS; HYOU1
clindamycin HL60 DOWN	7.75 × 10^−11^	TRAP1; PDXK; PITRM1; PRDX1; CRYZL1; ASNS; TALDO1; PAK2
Copper sulfate CTD 00007279	8.72 × 10^−10^	TRAP1; PDXK; MVP; ANXA5; ASNS; TALDO1; ACTG1; KLC4; HSPH1; PITRM1; TST; G3BP1; CRYZL1; BLVRB; PAK2; EIF4E
tanespimycin SKMEL5 UP	9.42 × 10^−11^	HSPH1; ASNS

**Table 5:** Enrichment analysis of the identified proteins by Enrichr software reporting molecular pathways by the BioPlanet2019 database, with relative *p*-values and gene names of differential proteins related to the terms.

Term	*p*-Value	Genes
Eukaryotic protein translation	0.002	EEF2; EIF4E
TGF-beta signaling pathway	0.002	TRAP1; STRAP; PAK2
Translation factors	0.002	EEF2; EIF4E
HIV-1 Nef as negative effector of Fas and TNF	0.003	PAK2; ACTG1
Signaling events mediated by hepatocyte growth factor receptor (c-Met)	0.005	PAK2; EIF4E
Unfolded protein response	0.005	ASNS; HYOU1
Sulfide oxidation to sulfate	0.006	TST
Heme degradation	0.006	BLVRB
Vitamin B6 metabolism	0.008	PDXK
eIF4E release	0.008	EIF4E

## Data Availability

Supporting proteomics data are available via ProteomeXchange with the identifier PXD027074.

## Supplementary Materials

The following are available online at https://www.mdpi.com/article/10.3390/toxics10010009/s1. Figure S1. Master gel of the three analyzed conditions. Red numbers and arrows indicate the differentially abundant spots among CTRL and DxCS. Differences are also reported in the gel obtained from DR-CALUX® treated with DMSO in order to highlight the same behavior between CTRL and DMSO protein spots. Table S1. PCDD/Fs parameters for acquisition in MS/MS mode, Table S2. PCB parameters for acquisition in MS/MS mode, Table S3. PCDD/Fs isotopic dilution and internal standard method, Table S4. PCB isotopic dilution and internal standard method, Table S5. PAH parameters for acquisition in MS/MS mode, Table S6. Pesticide parameters for acquisition in MS/MS mode, Table S7. Molecular lipophilicity of compounds found after Enrich analysis described by the partition coefficient log P. Data reported from DrugBank (https://go.drugbank.com/ (accessed on 1 October 2021)). Table S8. Statistical analysis of differential spots by Kruskal–Wallis and Dunn’s tests with z-value, *p*-value, *p*-adjusted, and the %V means ratio between the conditions. Table S9. MALDI-ToF mass spectrometry analysis. The table reports the 26 identified proteins with the spot number, protein name, UniProt name, UniProt and/or NCBI accession number, gene names, accession numbers of similar human proteins, the identity between Homo sapiens and Rattus/Mus musculus species, theoretical pI and MW, and Mascot Search Results (score, matched peptides, and coverage %).
