# Supplementary material for: First Attempt to Couple Proteomics with the AhR Reporter Gene Bioassay in Soil Pollution Monitoring and Assessment
Source: Toxics. 2021 Dec 29;10(1):9. doi: 10.3390/toxics10010009 (PMC8779689; doi:10.3390/toxics10010009)
Supplement: Supplementary file 1 [file toxics-10-00009-s001.zip › toxics-1500914-supplementary.pdf]

toxics

MDPI

Supplementary Materials: First Attempt to Couple Proteomic with *Ahr* Reporter Gene Bioassay in Soil Pollution Monitoring and Assessment

Claudia Landi, Giulia Liberatori, Pietro Cotugno, Lucrezia Sturba, Maria Luisa Vannuccini, Federica Massari, Daniela Valeria Miniero, Angelo Tursi, Enxhi Shaba, Peter Alexander Behnisch, Alfonso Carleo, Fabrizio Di Giuseppe, Stefania Angelucci, Luca Bini and Ilaria Corsi

Soil sample extraction and GC-MS/MS analysis of Dioxins, Furans and PCBs

A freeze-drying procedure was performed before soil sample extraction. Briefly, an amount of 80 g of fresh soil was lyophilized for 48 h (Labogene, Scanvac cool safe) using a pressure and temperature of respectively 0.5 torr and −50°C, in order to achieve a constant weight value. To remove the higher grain-size particles, the sample was sieved (2 mm), and this fraction was stored in a HDPE container until the chemical analysis. Seventeen 2,3,7,8-substituted PCDD/Fs (7 PCDDs and 10 PCDFs) and *dl*-PCBs were analyzed by gas-chromatography (GC Trace 1300, Thermo Scientific) coupled to mass-spectrometry (TSQ 8000, Triple Quadrupole, Thermo Scientific) using different GC programs under optimized detection conditions (Table S1 and S2). Isotope dilution method was applied for the quantitation according to 1613B and 1668C EPA methods. For this aim, before extraction, soil sample was fortified with a proper amount of labelled standard compounds, a mixture of $^{13}C_{12}$- PCDD/Fs and $^{13}C_{12}$-PCBs, to evaluate the recovery (Table S3 and S4). GC-MS/MS analysis of a sample was accepted when the recovery of the labelled compounds resulted in the range reported in EPA methods.

Sample extraction was performed by means of a pressurized liquid extraction technique, PLE, using an Accelerated Solvent Extractor (Dionex ASE 350, Thermo Scientific). Five grams of soil sample were subjected to ASE with hexane as solvent, and pre-mixed 1:1 with Dionex Prep DE, as sample dispersion agent. ASE method required the following conditions: three static cycles (10 minutes each) at 100 °C, 1500 psi pressure, 60 % of rinse volume and 100 seconds purge time with nitrogen flow. Labelled standards, purchased from Cambridge Isotope Laboratories (Andover MA, USA) and Cerilliant (Round Rock TX, USA), were used according to 1613B and 1668C EPA methods to evaluate the recovery of the analytes through the whole analytical method. Before extraction, labelled solutions at a proper concentration (100 ng/mL for $^{13}C_{12}$- PCDD/Fs and $^{13}C_{12}$-PCBs, and 200 ng/mL for $^{13}C_{12}$-OCDD) were added to soil samples. The extract was purified and fractioned by means of an automated clean-up system, called PowerPrep (Fluid Management System, Waltham, Massachusetts) using disposable columns (multilayer silica, alumina and carbon) and a multi-solvent switching programme. Two aliquots containing PCDD/Fs and PCBs, were obtained after clean-up and ripartition, and were then concentrated under a gentle nitrogen flow near dryness. Finally, 20 μL of labelled internal standard solutions at 100 ng/mL ($^{13}C_{12}$-PCDD/Fs and $^{13}C_{12}$-PCBs) were spiked to each vial before GC/MS analysis. 5 μL and 3 μL of standard solutions or sample were injected inside the heated PTV injector of the GC/MS instrument by means of a TriPlus RSH autosampler (Thermo Scientific) for the analysis of respectively PCDD/Fs and PCBs. Seventeen 2,3,7,8-substituted CDDs/CDFs (7 PCDDs and 10 PCDFs) and *dioxin-like* PCBs were separately analysed by gas-chromatography (GC Trace 1300, Thermo Scientific) coupled to mass-spectrometry (TSQ 8000, Triple Quadrupole, Thermo Scientific) using different GC programmes and under optimized detection conditions. For the separation of dioxins and *polychlorinated biphenyls* congeners, a Trace Gold TG-5MS capillary column (60 m × 0.25 mm, 0.25 μm film thickness, Thermo Scientific) was used. PTV injector, operating in large volume mode, was set at 130 °C, heated to 300 °C at 10.0 °C/sec during the transferring step, and further

heated to 320 °C for the cleaning after the passage of analytes into column. Oven temperature programme for PCDD/Fs was the following: for 1.0 min at 100 °C, from 100 °C to 200 °C (hold time 1.0 min) at 20.0 °C/min, from 200 °C to 290 °C (hold time 10.0 min) at 3.0 °C/min and from 290 °C to 320 °C (hold time 10.0 min) at 20.0 °C/min. For PCBs, oven temperature programme was originally set to 100 °C for 1.0 min, heated from 100 °C to 200 °C (hold time 1.0 min) at 20.0 °C/min, from 200 °C to 290 °C (hold time 5.0 min) at 2.5 °C/min and from 290 °C to 320 °C (hold time 5.0 min) at 30.0 °C/min. The transfer line and the ion source temperatures were set to 280 °C. Mass-spectrometry determinations were carried out operating with electron ionization (EI) at 45 eV in selected reaction monitoring (SRM) mode. Details about the m/z ⍰ m/z transitions for each native and surrogate congeners were reported in Table S1 and Table S2. For quantitation, calibration curves were constructed for PCDD/Fs and *dl*-PCBs. Five ready-to-use standard solutions in nonane (Cerilliant), in the concentration range 0.5-200 ng/mL (except for 2,3,7,8-TetraCDD/F, 0.1–40 ng/mL and OctaCDD/F, 1–400 ng/mL) were used to quantify PCDD/Fs according to 1613B EPA method. For PCBs, five standard solutions in nonane were prepared in the concentration range 0.2–400 ng/mL according to 1668C EPA method. Each calibration solution also contained labelled internal and recovery standards at 100 ng/mL. To evaluate the linearity of the calibration curves, the coefficient of correlation for each analyte, R^2 was calculated and it resulted to be always higher than 0.998. The qualitative analysis of *dioxins*, *furans* and *PCBs* in GC-MS/MS required to observe the absolute and relative retention times by comparison of standard chromatographic runs; two MS/MS transitions at a proper collision energy were set for MS acquisition and both were detected for each analyte, because the two product ions represent respectively the quantification and qualification ion. The applied method for quantitation is called Isotope Dilution and it provides for adding a known amount of labelled compounds to every sample prior to extraction. By using this approach, the correction for the recovery of PCDD/Fs and PCBs can be made because each analyte and its labelled compound exhibit similar properties during extraction, clean-up, concentration and gas-chromatography analysis. For quantitative determination, response ratios were used in conjunction with initial calibration data. Details about the quantitation of PCDD/Fs and PCBs by the isotope dilution method were reported in Tables S3 and S4. In particular, 1,2,3,7,8,9-HxCDD ($^{13}C_{12}$) was used as an instrument internal standard and therefore it was not used to quantitate the 1,2,3,7,8,9-HxCDD by means of standard isotope dilution method. For this reason, 1,2,3,7,8,9-HxCDD can be quantitated using the response of the other two labelled analogues 2,3,7,8-substituted HxCDD's: 1,2,3,4,7,8-HxCDD or 1,2,3,6,7,8-HxCDD. Moreover, the concentration of OCDF was corrected for the recovery of the labelled OCDD, $^{13}C_{12}$-OCDD, since its labelled analogue was not present in the labelled extraction standard mixture. For the congeners of *dl-PCBs*, the correction for the recovery was performed according to isotope dilution method, by using the response of the labelled analogue.

GC-MS/MS analysis of a sample was accepted when the recovery of the labelled standard compounds resulted in the range reported in EPA methods. Quality checks were performed according to EPA methods (1613B and 1668C) before starting the analysis on real samples. At the beginning of each 12-hour shift, GC/MS system performance and calibration were verified for all PCDD/Fs, PCBs and labelled compounds, by analysing the calibration verification (VER) standards, known as CS3, to verify performance criteria reported in EPA methods. Subsequently, one or more instrumental blanks (injection of hexane) and a blank sample were analysed by GC-MS/MS to evaluate freedom of contamination both instrumental system (carryover of the VER analysis) and during sample preparation procedure. To evaluate the recovery of each compounds, native and standard congeners, and verify the maintaining of the performance criteria reported in EPA methods (1613B and 1668C), inside the analysed batch, an OPR, Ongoing Precision and Recovery blank sample was prepared fortifying it with the same extraction standard mixtures of PCDD/Fs and PCBs ($^{13}C_{12}$ labeled) and a proper amount of mixture containing each native

congeners of PCDD/Fs and PCBs. This OPR sample was then submitted to the whole analytical procedure. For the native pollutants, the test concentration for OPR analysis was 10 ng/mL for 2,3,7,8- TCDD and 2,3,7,8- TCDF, 100 ng/mL for OCDD/F, 50 ng/mL for the other congeners of PCDD/Fs and for native PCBs.

Table S1. PCDD/Fs parameters for acquisition in MS/MS mode.

Native and Labeled Congener	m/z → m/z Transition
2,3,7,8-TCDF	303.9 – 240.9<br>305.9 – 242.9
2,3,7,8-TCDF ($^{13}C_{12}$)	315.9 – 252.0<br>317.9 – 254.0
1,2,3,4-TCDD ($^{13}C_{12}$)	331.9 – 268.0<br>333.9 – 270.0
2,3,7,8-TCDD	319.9 – 256.9<br>321.9 – 258.9
2,3,7,8-TCDD ($^{13}C_{12}$)	331.8 – 268.0<br>333.9 – 270.0
1,2,3,7,8-PCDF	337.9 – 274.9<br>339.9 – 276.9
1,2,3,7,8-PCDF ($^{13}C_{12}$)	349.9 – 285.9<br>351.9 – 287.9
2,3,4,7,8-PCDF	337.9 – 274.9<br>339.9 – 276.9
2,3,4,7,8-PCDF ($^{13}C_{12}$)	349.9 – 285.9<br>351.9 – 287.9
1,2,3,7,8-PCDD	353.9 – 290.9<br>355.9 – 292.9
1,2,3,7,8-PCDD ($^{13}C_{12}$)	365.9 – 301.9<br>367.9 – 303.9
1,2,3,4,7,8-HxCDF	371.8 – 308.9<br>373.8 – 310.9
1,2,3,4,7,8-HxCDF ($^{13}C_{12}$)	383.9 – 319.9<br>385.9 – 321.9
1,2,3,6,7,8-HxCDF	371.8 – 308.9<br>373.8 – 310.9
1,2,3,6,7,8-HxCDF ($^{13}C_{12}$)	383.9 – 319.9<br>385.9 – 321.9
1,2,3,7,8,9-HxCDF	371.8 – 308.9<br>373.8 – 310.9
1,2,3,7,8,9-HxCDF ($^{13}C_{12}$)	383.9 – 319.9<br>385.9 – 321.9
1,2,3,4,7,8-HxCDD	389.8 – 326.9<br>391.8 – 328.9
1,2,3,4,7,8-HxCDD ($^{13}C_{12}$)	399.9 – 335.9<br>401.9 – 337. 9
1,2,3,6,7,8-HxCDD	389.8 – 326.9<br>387.8 – 324.9
1,2,3,6,7,8-HxCDD ($^{13}C_{12}$)	399.9 – 335.9<br>401.9 – 337. 9
1,2,3,7,8,9-HxCDD	387.8 – 324.9

	389.8 – 326.9
1,2,3,7,8,9-HxCDD ($^{13}C_{12}$)	399.9 – 335.9<br>401.9 – 337. 9
2,3,4,6,7,8-HxCDF	373.8 – 310.9<br>371.8 – 308.9
2,3,4,6,7,8-HxCDF ($^{13}C_{12}$)	383.9 – 319.9<br>385.9 – 321.9
1,2,3,4,6,7,8-HpCDF	407.8 – 344.8<br>409.8 – 346.8
1,2,3,4,6,7,8-HpCDF ($^{13}C_{12}$)	419.8 – 355.9<br>421.8 – 357.9
1,2,3,4,6,7,8-HpCDD	423.8 – 360.8<br>425.8 – 362.8
1,2,3,4,6,7,8-HpCDD ($^{13}C_{12}$)	435.8 – 371.9<br>437.9 – 373.9
1,2,3,4,7,8,9-HpCDF	407.9 – 344.8<br>409.8 – 346.8
1,2,3,4,7,8,9-HpCDF ($^{13}C_{12}$)	419.8 – 355.9<br>421.8 – 357.9
OCDD	457.7 – 394.8<br>459.7 – 396.8
OCDD ($^{13}C_{12}$)	469.8 – 405.8<br>471.8 – 407.8
OCDF	441.8 – 378.8<br>443.8 – 380.8

Table S2. PCBs parameters for acquisition in MS/MS mode.

**Native and Labeled Congener**	**m/z→ m/z Transition**
TetraCBs (PCB 81, 77)	220.0 – 150.1<br>289.9 – 220.1
TetraCB ($^{13}C_{12}$)	302.0 – 232.1<br>304.0 – 234.1
PentaCBs (PCB 123, 118, 114, 105, 126)	323.9 – 254.0<br>325.9 – 256.0
PentaCB ($^{13}C_{12}$)	336.0 – 266.1<br>338.0 – 268.0
HexaCBs (PCB 167, 156, 157, 169)	289.9 – 220.0<br>359.9 – 290.0
HexaCB ($^{13}C_{12}$)	361.9 – 290.0<br>371.9 – 302.0
EptaCBs (PCB 189)	393.9 – 323.9<br>395.9 – 326.0
EptaCB ($^{13}C_{12}$)	405.9 – 336.0<br>407.7 – 336.0

Table S3. PCDD/Fs isotopic dilution and internal standard method.

**PCDD/Fs Method**		
Native compounds	Surrogate standard	Internal standard
2,3,7,8-TCDF	2,3,7,8-TCDF ($^{13}C_{12}$)	1,2,3,4-TCDD ($^{13}C_{12}$)

2,3,7,8-TCDD	2,3,7,8-TCDD ($^{13}C_{12}$)	1,2,3,4-TCDD ($^{13}C_{12}$)
1,2,3,7,8-PCDF	1,2,3,7,8-PCDF ($^{13}C_{12}$)	1,2,3,4-TCDD ($^{13}C_{12}$)
2,3,4,7,8-PCDF	2,3,4,7,8-PCDF ($^{13}C_{12}$)	1,2,3,4-TCDD ($^{13}C_{12}$)
1,2,3,7,8-PCDD	1,2,3,7,8-PCDD ($^{13}C_{12}$)	1,2,3,4-TCDD ($^{13}C_{12}$)
1,2,3,4,7,8-HxCDF	1,2,3,4,7,8-HxCDF ($^{13}C_{12}$)	1,2,3,7,8,9-HxCDD ($^{13}C_{12}$)
1,2,3,6,7,8-HxCDF	1,2,3,6,7,8-HxCDF ($^{13}C_{12}$)	1,2,3,7,8,9-HxCDD ($^{13}C_{12}$)
1,2,3,7,8,9-HxCDF	1,2,3,7,8,9-HxCDF ($^{13}C_{12}$)	1,2,3,7,8,9-HxCDD ($^{13}C_{12}$)
1,2,3,4,7,8-HxCDD	1,2,3,4,7,8-HxCDD ($^{13}C_{12}$)	1,2,3,7,8,9-HxCDD ($^{13}C_{12}$)
1,2,3,6,7,8-HxCDD	1,2,3,6,7,8-HxCDD ($^{13}C_{12}$)	1,2,3,7,8,9-HxCDD ($^{13}C_{12}$)
1,2,3,7,8,9-HxCDD	1,2,3,6,7,8-HxCDD ($^{13}C_{12}$)	1,2,3,7,8,9-HxCDD ($^{13}C_{12}$)
2,3,4,6,7,8-HxCDF	2,3,4,6,7,8-HxCDF ($^{13}C_{12}$)	1,2,3,7,8,9-HxCDD ($^{13}C_{12}$)
1,2,3,4,6,7,8-HpCDF	1,2,3,4,6,7,8-HpCDF ($^{13}C_{12}$)	1,2,3,7,8,9-HxCDD ($^{13}C_{12}$)
1,2,3,4,6,7,8-HpCDD	1,2,3,4,6,7,8-HpCDD ($^{13}C_{12}$)	1,2,3,7,8,9-HxCDD ($^{13}C_{12}$)
1,2,3,4,7,8,9-HpCDF	1,2,3,4,7,8,9-HpCDF ($^{13}C_{12}$)	1,2,3,7,8,9-HxCDD ($^{13}C_{12}$)
OCDD	OCDD ($^{13}C_{12}$)	1,2,3,7,8,9-HxCDD ($^{13}C_{12}$)
OCDF	OCDD ($^{13}C_{12}$)	1,2,3,7,8,9-HxCDD ($^{13}C_{12}$)

Table S4. PCBs isotopic dilution and internal standard method.

**PCBs Method**		
Native compounds	Surrogate standard	Internal standard (IS)
PCB 81 (*dl*)	PCB 81 ($^{13}C_{12}$)	PCB 111 ($^{13}C_{12}$)
PCB 77 (*dl*)	PCB 77 ($^{13}C_{12}$)	PCB 111 ($^{13}C_{12}$)
PCB 123 (*dl*)	PCB 123 ($^{13}C_{12}$)	PCB 111 ($^{13}C_{12}$)
PCB 118 *(dl)*	PCB 118 ($^{13}C_{12}$)	PCB 111 ($^{13}C_{12}$)
PCB 114 *(dl)*	PCB 114 ($^{13}C_{12}$)	PCB 111 ($^{13}C_{12}$)
PCB 105 *(dl)*	PCB 105 ($^{13}C_{12}$)	PCB 111 ($^{13}C_{12}$)
PCB 126 *(dl)*	PCB 126 ($^{13}C_{12}$)	PCB 111 ($^{13}C_{12}$)
PCB 167 *(dl)*	PCB 167 ($^{13}C_{12}$)	PCB 111 ($^{13}C_{12}$)
PCB 156 *(dl)*	PCB 156 ($^{13}C_{12}$)	PCB 170 ($^{13}C_{12}$)
PCB 157 *(dl)*	PCB 157 ($^{13}C_{12}$)	PCB 170 ($^{13}C_{12}$)
PCB 169 *(dl)*	PCB 169 ($^{13}C_{12}$)	PCB 170 ($^{13}C_{12}$)
PCB 189 *(dl)*	PCB 189 ($^{13}C_{12}$)	PCB 170 ($^{13}C_{12}$)

Soil sample extraction and GC-MS/MS analysis of Polycyclic Aromatic Hydrocarbons and pesticides

In the present study, Polycyclic Aromatic Hydrocarbons (PAHs), some organochloride pesticides and a herbicide were determined. The Italian National Regulation, D. Lgs. 152/2006, required to evaluate in soil samples the presence of these PAHs and to report the sum of some of them: pyrene, benzo(a)anthracene, chrysene, benzo(b)fluoranthene, benzo(k)fluoranthene, benzo(a)pyrene, indeno(1,2,3-c,d)pyrene, dibenzo(a,h)anthracene, benzo(g,h,i)perylene, dibenzo(a,l)pyrene, dibenzo(a,i)pyrene, dibenzo(a,e)pyrene and dibenzo(a,h)pyrene. Analytical method for phytochemicals was optimized for alachlor, aldrin, dieldrin, atrazine, chlordane, α, ß, γ isomers of exachlorocyclohexane, *p,p'*-DDT, *p,p'*-DDE and *p,p'*-DDD. The extraction of PAHs and pesticides was performed in one step by ASE (Dionex ASE 350, Thermo Scientific), using a mixture of hexane/dichloromethane (1/1, v/v) as extracting solvent. Five grams of freeze-dried sample were subjected to ASE by means of these operating conditions: temperature of 100°C, pressure of 1500 psi, one static cycle of 5 min, rinse volume of 30%, purge time with nitrogen flow equal to 60 seconds. The extracted sample, after solvent evaporation, was purified by a Gel Permeation Chromatography System (Knauer, Germany), using a glass column (50 mm length, 10 mm

internal diameter) packed with a proper amount of a pre-swelled styrene-divinylbenzene copolymer. A mixture of cyclohexane/dichloromethane (70/30, v/v) was used as mobile phase, at a flow rate of 1 mL/min. During the chromatographic run, the fraction eluting from GPC in the range time between 18 min and 48 min was collected and evaporated under a gentle nitrogen flow. Then, 475 µL of hexane and 25 µL of internal standard solution (initial concentration of 1 mg/L) were added to the concentrated sample for GC-MS analysis. The identification and quantification of PAHs and pesticides were performed by means of a Gas-Chromatograph (7890B, Agilent), with a split/splitless injector, coupled to a Mass Spectrometer (7000B, Triple Quadrupole, Agilent) in two different chromatographic runs. A gas-chromatographic column Trace Gold TG-SQC (30 m × 0.25 mm, 0.25µm film thickness, Thermo Scientific) was used for the separation. The S/SL injector was used in splitless mode at 280 °C. 2 µL of standard solution or sample were injected by means of the autosampler. The optimized temperature program for PAHs and pesticides was the following: 80 °C for 3 min, from 80 °C to 300 °C at 15 °C/min, 300 °C for 15 min. The temperature of transfer line was set to 300 °C, the ion source (EI mode, 70 eV) and quadrupole temperatures were set respectively to 280 °C and 150 °C. The acquisition of each analyte and standard compound was performed in selected reaction monitoring, SRM, using the response of two product ions for each analyte (quantitation and qualification ion, m/z). Detailed information about the m/z → m/z transitions was reported in Tables S5 and S6. Calibration curves for PAHs and phytochemicals were obtained using five diluted standard solutions from stock mixtures, supplied by Ultrascientific (Italy). They resulted linear (R^2 higher than 0.997) in the concentration range between 10 ng/mL and 200 ng/mL for PAHs, and between 5 ng/mL to 200 ng/mL for pesticides.

The quantification of each compound was performed by internal standard method according to the 8270 EPA method, using the response of acenaphthene-d_{10}, phenanthrene-d_{10}, chrysene-d_{12} and perylene-d_{12} (IS). As descripted for PCDD/Fs and PCBs, the recovery of PAHs and pesticides was evaluated using a similar approach: a surrogate standard mixture, containing 2-fluorobiphenyl and *p*-terphenyl-d_{14} was added to the sample before extraction according to 8270 EPA method. Recovery values for the extraction standards in the range between 70–120% were considered acceptable.

Table S5. PAHs parameters for acquisition in MS/MS mode.

PAHs and Surrogate Compounds	m/z→ m/z Transition
2-fluorobiphenyl	172.0 – 171.0
	170.0 – 169.0
pyrene	202.1 – 201.1
	200.1 – 199.2
p-terphenyl-d_{14}	244.2 – 242.2
	244.2 – 240.2
chrysene	228.1 – 226.1
	226.1 – 224.1
benzo(a)anthracene	228.1 – 226.1
	226.1 – 224.1
benzo(b)fluoranthene	250.1 – 248.1
	250.1 – 249.1
benzo(k)fluoranthene	252.1 – 250.1
	250.1 – 248.1
benzo(a)pyrene	250.1 – 248.2
	250.1 – 249.2
indeno(1,2,3-c,d)pyrene	275.9 – 273.9
	273.9 – 271.9
dibenzo(a,h)anthracene	277.9 – 275.9

	277.9 – 276.9
benzo(g,h,i)perylene	275.9 – 273.9<br>273.9 – 271.9
dibenzo(a,l)pyrene	302.2 – 300.2<br>300.2 – 298.2
dibenzo(a,i)pyrene	302.2 – 300.2<br>300.2 – 298.2
dibenzo(a,h)pyrene	302.2 – 300.2<br>300.2 – 298.2
dibenzo(a,e)pyrene	302.2 – 300.2<br>300.2 – 298.2
chrysene-d_{12} (IS)	240.2 – 238.2<br>236.1 – 232.2
perylene-d_{12} (IS)	264.2 – 260.2<br>260.2 – 256.2

Table S6. Pesticides parameters for acquisition in MS/MS mode.

Pesticides and Surrogate Compounds	m/z→ m/z Transition
2-fluorobiphenyl	172.0 – 171.0<br>170.0 – 169.0
acenaphthene-d_{10} (IS)	162.2 – 160.2<br>164.2 – 162.2
α -HCH	218.8 – 108.9<br>218.8 – 183.0
atrazine	214.9 – 199.9<br>214.9 – 172.9
γ -HCH	218.8 – 108.9<br>218.8 – 183.0
ß -HCH	218.8 – 108.9<br>218.8 – 183.0
phenanthrene-d_{10} (IS)	188.2 – 160.1<br>188.2 – 184.2
alachlor	187.9 – 145.9<br>187.9 – 159.9
aldrin	262.7 – 192.9<br>262.7 – 190.9
chlordane	372.9 – 262.8<br>372.9 – 236.8
p,p′-DDE	245.8 – 175.8<br>317.7 – 245.8
p-terphenyl-d_{14}	244.2 – 242.2<br>244.2 – 240.2
dieldrin	262.7 – 192.9<br>79.0 – 77.0
p,p′-DDD	234.8 – 164.9<br>236.8 – 164.9
p,p′-DDT	234.8 – 164.9<br>236.8 – 164.9
chrysene-d_{12} (IS)	240.2 – 238.2<br>236.1 – 232.2

Soil sample extraction and GC-FID analysis of C > 12 Hydrocarbons

The determination of C > 12 Hydrocarbons was performed according to the procedure No 75/2011 reported by ISPRA, Institute for Environmental Protection and Research, an important Italian institution conducting scientific research in the environmental area (Procedura per l'analisi degli idrocarburi >C_{12} in suoli contaminati, Manuali e Linee Guida, n. 75/2011, available at https://www.isprambiente.gov.it/contentfiles/00010400/10425-mlg-75-2011.pdf/).

Ten grams of freeze-dried soil sample, pre-mixed with Dionex Prep DE, were extracted by the reported ASE (Dionex ASE 350, Thermo Scientific) method, using a mixture of hexane/acetone (1/1, v/v): temperature of 100 °C, pressure of 1500 psi, two static cycles (5 min each), rinse volume of 30%, purge time with nitrogen equal to 60 seconds. After extraction, the sample was concentrated and resumed in 10 mL of the RTW (retention time window) solution, containing n-dodecane and n-tetracontane. This solution was then loaded onto a SPE column, Supelclean™ Florisil/Na_2SO_4 (Sigma Aldrich, Italy) for the cleanup. An aliquot of eluted fraction was then directly analyzed by GC-FID, using a Gas-chromatograph (7890B Agilent), equipped with a split/splitless injector and a flame ionization detector. A dedicated gas-chromatography capillary column, HT5 column (5% Phenyl, Polycarborane-siloxane, 25 m × 320 μm, 0.1 μm film thickness, SGE-Analytical Science) was used for separation with the following temperature program: 80°C for 1 min; from 80 °C to 320 °C at 20 °C/min; 320 °C for 1 min; from 320 °C to 380 °C at 20 °C/min; 380 °C for 10 min. A split/splitless injector was set in splitless mode at 270 °C. For the detection of heavy hydrocarbons, C > 12, the flame ionization detector, FID, was set at 280 °C with a flame obtained from a mixture hydrogen/air (flow rate for hydrogen 30.0 mL/min, flow rate for air 400 mL/min). For quantification, a calibration curve was constructed using a standard solution in heptane containing two types of mineral oil, A and B, and the RTW solution. The linearity of the analytical method was explored in the concentration range between 30 and 150 mg/Kg. The integration of the chromatogram takes into account the time window delimited by the two hydrocarbons, n-dodecane and n-tetracontane. Indeed, ISPRA procedure requires, for the integration of a chromatogram, to begin immediately after the end of n-dodecane peak and to end before the beginning of the n-tetracontane peak.

Table S7. Molecular lipophilicity of compound found after Enrich analysis described by the partition coefficient Log P. Data reported from DrugBank (https://go.drugbank.com/).

**Compounds**	**Molecular Lipophilicity (Log P)**	**Hydrophobic/Hydrophilic**
Vorinostat	1.88 – 2	Hydrophobic
Troglitazone	4.16 – 5.5	Hydrophobic
Chlortetracycline	−0.13 – −2.9	Hydrophilic
Lobeline	3.73 – 3.78	Hydrophobic
Clonidine	2.49 – 2.55	Hydrophobic
Potassium dichromate	−0.61 – −3.7	Hydrophilic
Atrazine	2.2 – 2.7	Hydrophobic
Clindamycin	1.04 – 1.76	Hydrophobic
Copper sulfate	−0.84	Hydrophilic
Tanespimicin	1.52 – 2.53	Hydrophobic
Thapsigargin	5.102	Hydrophobic
Lomustine	2.16 – 2.62	Hydrophobic
Fluorouracil	−0.58 – −0.66	Hydrophilic
Glibenclamid	3.78	Hydrophobic
Cyclosporine	1.4	Hydrophobic
Chlorpromazine	4.54 – 5.18	Hydrophobic

Imipramine	4.8	Hydrophobic
Desipramine	4.9	Hydrophobic
Fluoxetine	4.05	Hydrophobic
Chlorprothixene	5.07 – 5.42	Hydrophobic

Table S8. Statistical analysis of differential spots by Kruskal Wallis and Dunn's test with z-value, *p*-value and p-adjusted and the %V means ratio between the conditions, were reported. Not valid statistical values were shown in red.

		DxCS		DMSO		Ctrl		Kruskal wallis pvalue	Dunn´s Multiple Comparison Test														
															DxCS vs DMSO			DxCS vs Ctrl			DMSO vs Ctrl		
**Spot n**	**Protein Name**	**Mean**	**SD**	**Mean**	**SD**	**Mean**	**SD**		**DxCS/DMSO**	**DMSO/DxCS**	**DxCS/Ctrl**	**Ctrl/DxCS**	**DMSO/Ctrl**	**ctrl/DMSO**	**Z-value**	**P.value**	**p.adj**	**Z-value**	**P.value**	**p.adj**	**Z-value**	**P.value**	**p.adj**
**1**		0,02039	0,00368	0,00864	0,00596	0,00919	0,00168	4,55E-02	**2,359786737**	0,423767108	**2,220111**	0,450428	0,9408099	1,0629139	2,37	2,42E-02	3,63E-02	1,97	8,93E-03	2,68E-02	0,43	3,35E-01	3,35E-01
**2**	Hypoxia up-regulated protein 1	0,06403	0,01344	0,04905	0,00781	0,02813	0,00539	1,98E-02	1,305551011	0,765960113	**2,276013**	0,439365	1,7433352	0,5736131	2,70	1,78E-01	1,78E-01	0,92	3,49E-03	1,05E-02	1,92	2,75E-02	4,13E-02
**3**	Phosphoribosyl formylglycinamidine synthase	0,03229	0,00418	0,03094	0,00833	0,01785	0,00269	3,02E-02	1,043658553	0,958167781	**1,808528**	0,552936	1,732873	0,5770764	2,17	5,00E-01	5,00E-01	0,00	1,50E-02	2,24E-02	2,35	9,51E-03	2,85E-02
**4**		0,01084	0,00337	0,00417	0,00295	0,00538	0,00242	4,06E-02	**2,600125426**	0,384596831	**2,015324**	0,496198	0,7750872	1,2901774	1,88	6,81E-03	2,04E-02	2,47	3,04E-02	4,56E-02	-0,64	2,61E-01	2,61E-01
**5**		0,01196	0,00188	0,00377	0,00475	0,0058	0,00173	4,00E-02	**3,170264189**	0,315431125	**2,061897**	0,48499	0,6503866	1,537547	1,88	6,70E-03	2,01E-02	2,47	3,01E-02	4,51E-02	-0,64	2,61E-01	2,61E-01
**6**		0,00495	0,00129	0,01058	0,00273	0,00409	0,00106	1,98E-02	0,467564475	**2,138742468**	1,208983	0,827141	**2,5857042**	0,3867418	0,92	5,00E-02	7,50E-02	-1,64	1,78E-01	1,78E-01	2,77	2,79E-03	8,37E-03
**7**	Ubiquitin-like modifier-activating enzyme 1	0,0788	0,01921	0,05933	0,00607	0,03131	0,01727	2,38E-02	1,328008699	0,753007116	**2,516467**	0,397383	**1,8949174**	0,5277275	2,57	2,45E-01	2,45E-01	0,69	5,14E-03	1,54E-02	2,03	2,14E-02	3,21E-02
**8**	heat shock protein 105 kDa	0,01613	0,00052	0,00735	0,00249	0,00714	0,00183	4,87E-02	**2,195928085**	0,455388319	**2,259725**	0,442532	1,0290523	0,971768	2,27	1,91E-02	2,87E-02	2,07	1,16E-02	3,48E-02	0,21	4,16E-01	4,16E-01
**9**	Major vault protein	0,06467	0,01923	0,0235	0,00209	0,02449	0,0034	4,87E-02	**2,751695463**	0,363412308	**2,640854**	0,378665	0,9597188	1,0419719	2,27	1,91E-02	2,87E-02	2,07	1,16E-02	3,48E-02	0,21	4,16E-01	4,16E-01
**10**	Pitrilysin metallepetidase 1 (Predicted)	0,04323	0,01074	0,00874	0,0112	0,01327	0,00582	4,00E-02	**4,946692225**	0,20215529	**3,258225**	0,306916	0,6586674	1,518217	1,88	6,70E-03	2,01E-02	2,47	3,01E-02	4,51E-02	-0,64	2,61E-01	2,61E-01
**11**	alanine--tRNA ligase, cytoplasmic	0,1835	0,02732	0,11159	0,0237	0,09416	0,01344	3,46E-02	1,644489044	0,608091616	**1,948823**	0,51313	1,185063	0,843837	2,57	3,78E-02	5,67E-02	1,78	5,14E-03	1,54E-02	0,85	1,97E-01	1,97E-01
**12**		0,05821	0,01068	0,02896	0,00829	0,02427	0,00177	3,46E-02	**2,009814186**	0,497558434	**2,398538**	0,416921	1,1934128	0,837933	2,57	3,78E-02	5,67E-02	1,78	5,14E-03	1,54E-02	0,85	1,97E-01	1,97E-01

**13**	elongation factor 2	0,0292	0,01137	0,14633	0,03891	0,15686	0,03459	4,87E-02	0,199581428	**5,010486254**	0,186189	**5,370897**	0,9328957	1,0719312	-2,27	1,91E-02	2,87E-02	-2,07	1,16E-02	3,48E-02	-0,21	4,16E-01	4,16E-01
**14**		0,0308	0,00758	0,01372	0,00268	0,01708	0,00274	2,82E-02	**2,244800425**	0,4454739	**1,803087**	0,554604	0,8032284	1,2449759	1,68	3,85E-03	1,16E-02	2,66	4,67E-02	7,00E-02	-1,07	1,43E-01	1,43E-01
**15**		0,00604	0,00066	0,00441	0,00311	0,00911	0,0016	2,38E-02	1,369210468	0,730347907	0,662405	**1,50965**	0,4837863	**2,0670283**	-1,78	2,45E-01	2,45E-01	0,69	3,78E-02	5,67E-02	-2,67	3,85E-03	1,15E-02
**16**		0,02679	0,00176	0,01553	0,0053	0,01258	0,00353	4,06E-02	**1,724732914**	0,579799917	**2,12913**	0,469675	1,2344695	0,8100646	2,47	3,04E-02	4,56E-02	1,88	6,81E-03	2,04E-02	0,64	2,61E-01	2,61E-01
**17**	Heat shock protein 75 kDa, mitochondrial	0,03981	0,00442	0,01941	0,01388	0,01463	0,00981	4,26E-02	**2,051269895**	0,487502889	**2,721404**	0,367457	1,3266923	0,7537543	2,42	2,69E-02	4,03E-02	1,93	7,69E-03	2,31E-02	0,53	2,97E-01	2,97E-01
**18**	kinesin light chain 4	0,02033	0,01585	0,01001	0,00155	0,00784	0,00127	4,09E-02	**2,031917341**	0,492146004	**2,594105**	0,385489	1,2766784	0,7832826	2,50	1,32E-01	1,32E-01	1,12	6,21E-03	1,86E-02	1,49	6,78E-02	1,02E-01
**19**		0	0	0	0	0,0076	0,00171	9,14E-03	**#DIV/0!**	**#DIV/0!**	0	**#DIV/0!**	0	**#DIV/0!**	-2,51	5,00E-01	5,00E-01	0,00	5,96E-03	8,93E-03	-2,72	3,30E-03	9,90E-03
**20**	ras GTPase-activating protein-binding protein 1	0,05269	0,00826	0,00676	0,00788	0,00656	0,00529	4,71E-02	**7,788090891**	0,128401172	**8,026662**	0,124585	1,0306329	0,9702776	2,19	1,42E-02	2,13E-02	2,19	1,42E-02	4,27E-02	0,00	5,00E-01	5,00E-01
**21**	myotonin-protein kinase isoform X1	0,03746	0,00259	0,02412	0,00865	0,01699	0,00587	3,46E-02	**1,552974712**	0,643925489	**2,205054**	0,453504	1,4198904	0,7042797	2,57	3,78E-02	5,67E-02	1,78	5,14E-03	1,54E-02	0,85	1,97E-01	1,97E-01
**22**		0,01793	0,00292	0,07226	0,02563	0,06393	0,00716	4,06E-02	0,248069246	**4,031132504**	0,280404	**3,566277**	1,1303475	0,8846837	-1,88	6,81E-03	2,04E-02	-2,47	3,04E-02	4,56E-02	0,64	2,61E-01	2,61E-01
**23Q**		0	0	0,02188	0,00621	0,02854	0,00548	3,25E-02	0	**#DIV/0!**	0	**#DIV/0!**	0,7667187	1,3042593	-2,59	3,65E-02	5,47E-02	-1,79	4,80E-03	1,44E-02	-0,86	1,95E-01	1,95E-01
**24**	Asparagine synthetase [glutamine-hydrolyzing]	0,02584	0,00427	0,04042	0,00982	0,05777	0,01624	3,08E-02	0,639415077	**1,563929342**	0,447385	**2,23521**	0,6996789	1,4292271	-2,63	8,87E-02	8,87E-02	-1,35	4,25E-03	1,27E-02	-1,39	8,29E-02	1,24E-01
**25**	serine/threonine-protein kinase PAK 2	0,08849	0,04938	0,02444	0,02437	0,02169	0,00251	4,55E-02	**3,621412077**	0,27613538	**4,079628**	0,24512	1,1265297	0,8876818	1,97	8,93E-03	2,68E-02	2,37	2,42E-02	3,63E-02	-0,43	3,35E-01	3,35E-01
**26**	Actin, cytoplasmic 2	0,07396	0,01018	0,02874	0,01035	0,02669	0,00728	4,55E-02	**2,572877392**	0,3886699	**2,771301**	0,360841	1,0771215	0,9284004	2,37	2,42E-02	3,63E-02	1,97	8,93E-03	2,68E-02	0,43	3,35E-01	3,35E-01
**27**		0,12694	0,00454	0,02546	0,017	0,03058	0,00283	4,06E-02	**4,985579082**	0,200578505	**4,150365**	0,240943	0,8324741	1,2012386	2,47	3,04E-02	4,56E-02	1,88	6,81E-03	2,04E-02	0,64	2,61E-01	2,61E-01
**28**		0,01946	0,00169	0,06843	0,02468	0,03685	0,02034	2,20E-02	0,284368724	**3,516561124**	0,52816	**1,893366**	1,8573063	0,5384142	-1,58	2,86E-03	8,58E-03	-2,76	5,72E-02	8,57E-02	1,28	1,00E-01	1,00E-01

**29**	quinone oxidoreductase-like protein 1	0,04527	0,00746	0,00914	0,00181	0,00877	0,00229	4,87E-02	**4,951841966**	0,201945055	**5,163253**	0,193676	1,0426934	0,9590547	2,27	1,91E-02	2,87E-02	2,07	1,16E-02	3,48E-02	0,21	4,16E-01	4,16E-01
**30**	serine-threonine kinase receptor-associated protein	0,04107	0,01523	0,08522	0,02108	0,08455	0,00622	4,87E-02	0,481883701	**2,075189508**	0,485693	**2,058915**	1,0079045	0,9921575	-2,07	1,16E-02	3,48E-02	-2,27	1,91E-02	2,87E-02	0,21	4,16E-01	4,16E-01
**31**	transaldolase	0,05358	0,01206	0	0	0,00542	0,00635	1,83E-02	**#DIV/0!**	0	**9,890472**	0,101107	0	#DIV/0!	1,94	2,57E-03	7,71E-03	2,80	2,64E-02	3,95E-02	-0,93	1,76E-01	1,76E-01
**32**		0,00409	0,00709	0,04506	0,01444	0,04808	0,02009	4,80E-02	0,09083283	**11,00923528**	0,085128	**11,74706**	0,9371905	1,0670189	-2,28	1,89E-02	2,83E-02	-2,08	1,14E-02	3,43E-02	-0,21	4,15E-01	4,15E-01
**33**	Pyridoxal kinase	0,01924	0,00718	0,02056	0,01397	0,04121	0,01225	4,63E-02	0,935510447	1,06893515	0,466815	**2,142176**	0,4989951	**2,0040277**	-2,20	3,71E-01	3,71E-01	-0,33	1,38E-02	4,13E-02	-2,03	2,14E-02	3,21E-02
**34**	thiosulfate sulfurtransferase	0,11323	0,02921	0,03887	0,02868	0,05642	0,01889	4,06E-02	**2,912717257**	0,343322029	**2,006847**	0,498294	0,6889949	1,4513897	1,88	6,81E-03	2,04E-02	2,47	3,04E-02	4,56E-02	-0,64	2,61E-01	2,61E-01
**35**	annexin A5 isoform X1	0,04335	0,01408	0	0	0,00836	0,00982	1,83E-02	**#DIV/0!**	0	**5,183369**	0,192925	0	#DIV/0!	1,94	2,57E-03	7,71E-03	2,80	2,64E-02	3,95E-02	-0,93	1,76E-01	1,76E-01
**36Q**		0,09498	0,02826	0	0	0,00172	0,00343	1,58E-02	**#DIV/0!**	0	**55,34739**	0,018068	0	#DIV/0!	2,29	3,04E-03	9,12E-03	2,74	1,11E-02	1,67E-02	-0,49	3,11E-01	3,11E-01
**37**		0,03529	0,01062	0,07961	0,00444	0,0855	0,01742	4,98E-02	0,443306468	**2,255775797**	0,412785	**2,422571**	0,9311494	1,0739415	-2,17	1,50E-02	2,24E-02	-2,17	1,50E-02	4,49E-02	0,00	5,00E-01	5,00E-01
**38**	flavin reductase (NADPH)	0,09793	0,00897	0,00706	0,00861	0,00817	0,0132	4,32E-02	**13,87086958**	0,072093533	**11,99014**	0,083402	0,8644119	1,1568559	2,22	1,31E-02	1,97E-02	2,22	1,31E-02	3,94E-02	0,00	5,00E-01	5,00E-01
**39**	glutathione S-transferase Mu 2	0,01557	0,0135	0,04652	0,00633	0,04623	0,00735	4,98E-02	0,33476689	**2,987153241**	0,336882	**2,968401**	1,0063174	0,9937223	-2,17	1,50E-02	2,24E-02	-2,17	1,50E-02	4,49E-02	0,00	5,00E-01	5,00E-01
**40**	Eukaryotic translation initiation factor 4E	0,04234	0,01404	0,01772	0,01241	0,02073	0,0027	4,87E-02	**2,389983043**	0,41841301	**2,042876**	0,489506	0,8547659	1,169911	2,27	1,91E-02	2,87E-02	2,07	1,16E-02	3,48E-02	0,21	4,16E-01	4,16E-01
**41**	peroxiredoxin-1	0,06445	0,00628	0,03901	0,0119	0,03082	0,00233	3,46E-02	1,652190724	0,605256999	**2,091021**	0,478235	1,2656051	0,7901359	2,57	3,78E-02	5,67E-02	1,78	5,14E-03	1,54E-02	0,85	1,97E-01	1,97E-01
**42**		0,02735	0,00128	0,00692	0,00573	0,00462	0,00381	4,26E-02	**3,951702125**	0,253055511	**5,921896**	0,168865	1,4985683	0,6673036	2,42	2,69E-02	4,03E-02	1,93	7,69E-03	2,31E-02	0,53	2,97E-01	2,97E-01
**43**	2-iminobutanoate/2-	0,01812	0,00376	0,03158	0,01219	0,04881	0,00904	4,09E-02	0,57371502	**1,743025656**	0,37118	**2,694109**	0,6469767	1,5456506	-2,50	1,32E-01	1,32E-01	-1,12	6,21E-03	1,86E-02	-1,49	6,78E-02	1,02E-01

iminopropanoate deaminase

Table S9. MALDI-ToF mass spectrometry analysis. Table reports the 26 identified proteins with the spot number, Protein name, UniProt name, UniProt and/or NCBI accession number, gene names, accession number of similar human proteins, the identity between *Homo sapiens* and *rattus/mus musculus* species, theoretical p*I* and MW and Mascot Search Results (Score, Matched Peptides and Coverage %).

Spot n	Protein Name	UniProt Name	UniProt/NCBI AC	Gene Names	Human Similarity	Identity	Theorethical pI/MW	Score	Matched Peptides	Coverage %
**2**	**Hypoxia up-regulated protein 1**	**HYOU1_RAT**	**Q63617**	**Hyou1**	**Q9Y4L1**	**91.7%**	**5.11 11448**	**200**	21	23
**3**	Phosphoribosylformylglycinamidine synthase	PUR4_MOUSE	Q5SUR0	Pfas	O15067	88.5%	5.43 146248	82	43	36
**7**	Ubiquitin-like modifier-activating enzyme 1	UBA1_RAT	Q5U300	Uba1	P22314	96.4%	5.36 118910	363	43	49
**8**	Heat shock protein 105 kDa	HS105_RAT	Q66HA8	Hsph1	Q92598	92.3%	5.40 97327	275	33	39
	Ubiquitin-like modifier-activating enzyme 1	UBA1_RAT	Q5U300	Uba1	P22314	96.4%	5.36 118910	104	19	24
**9**	Major vault protein	MVP_RAT	Q62667	Mvp	Q14764	91.5%	5.43 96081	185	25	35
**10**	Presequence protease, mitochondrial		NP_001100833.1	Pitrm1	Q5JRX3	83.4%	6.94 109559	112	45	44
	Pitrilysin metallepetidase 1 (Predicted)	D3ZUF9_RAT	D3ZUF9							
**11**	Alanine--tRNA ligase, cytoplasmic	SYAC_RAT	P50475	Aars1	P49588	95.1%	5.41 107522	259	48	48
	Heat shock protein 105 kDa	HS105_RAT	Q66HA8	Hsph1	Q92598	92.3%	5.40 97327	192	40	50
**13**	Elongation factor 2	EF2_RAT	P05197	Eef2	P13639	99.2%	6.41 96192	524	54	57
**17**	Heat shock protein 75 kDa, mitochondrial	TRAP1_RAT	Q5XHZ0	Trap1	Q12931	88.2%	6.56 80639	442	46	55
**18**	Kinesin light chain 4	KLC4_RAT	Q5PQM2	Klc4	Q9NSK0	95.8%	5.89 69376	579	47	64
**20**	Ras GTPase-activating protein-binding protein 1		NP_598249.1	G3bp1	Q13283	93.4%	5.41 51812	95	20	40
		G3BP1_MOUSE	P97855							

**21**	Myotonin-protein kinase isoform X1	 DMPK_MOUSE	XP_006223166.1<br>P54265	Dmpk	Q09013	86.3%	5.02 76780	40	10	26
**24**	Asparagine synthetase [glutamine-hydrolyzing]	ASNS_RAT	P49088	Asns	P08243	93.6%	6.01 64776	402	41	66
**25**	Serine/threonine-protein kinase PAK 2	PAK2_RAT	Q64303	Pak2	Q13177	96.9%	5.37 58209	265	26	42
**26**	Actin, cytoplasmic 2	ACTG_RAT	P63259	Actg1	P63261	100%	5.29 42052	220	19	42
**29**	Quinone oxidoreductase-like protein 1	 QORL1_MOUSE	NP_001013062.1<br>Q921W4	Cryzl1	O95825	90.5%	5.59 39044	143	27	65
**30**	Serine-threonine kinase receptor-associated protein	STRAP_RAT	Q5XIG8	Strap	Q9Y3F4	97.7%	4.99 38717	232	25	72
**31**	Transaldolase	TALDO_RAT	Q9EQS0	Taldo 1	P37837	94.7%	6.57 37608	312	27	51
**33**	Pyridoxal kinase	PDXK_RAT	O35331	Pdxk	O00764	85.3%	6.32 35114	255	33	68
**34**	Thiosulfate sulfurtransferase	THTR_RAT	P24329	Tst	Q16762	90.2%	7.71 33614	313	25	58
**35**	Annexin A5 isoform X1	 ANXA5_RAT	XP_006232330.1<br>P14668	Anxa5	P08758	91.8%	4.93 35807	136	12	41
**38**	Flavin reductase (NADPH)	BLVRB_MOUSE	NP_001099706.1<br>Q923D2	Blvrb	P30043	93.7%	6.29 22194	141	17	87
**39**	Glutathione S-transferase Mu 2	GSTM2_RAT	P08010	Gstm2	P09488	81.7%	6.90 25857	300	28	90
**40**	Eukaryotic translation initiation factor 4E	IF4E_RAT	P63074	Eif4e	P06730	98.2%	5.79 25266	134	12	40
**41**	Peroxiredoxin-1	PRDX1_RAT	NP_476455.1<br>Q63716	Prdx1	Q06830	97.5%	8.27 22323	184	13	47
**43**	2-iminobutanoate/2-iminopropanoate deaminase	RIDA_RAT	NP_113902.1<br>P52759	Rida	P52758	87.6%	6.21 14338	118	7	79

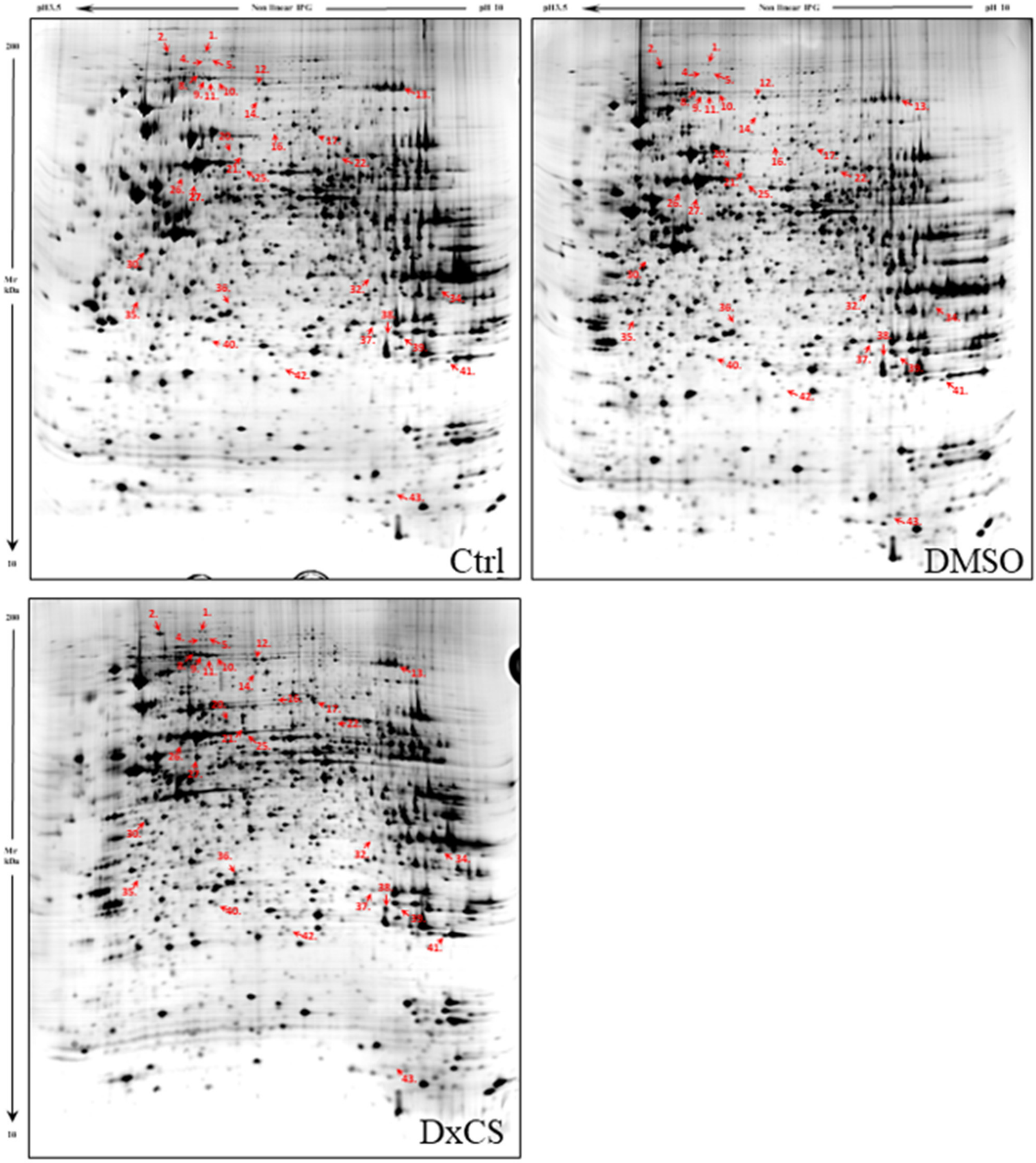

Figure S1. Master gel of the three analyzed conditions. Red numbers and arrows indicate the differentially abundant spots among Ctrl, and DxCS. Differences are also reported in the gel obtained from DR-CALUX® treated with DMSO in order to highlight the same behavior between Ctrl and DMSO protein spots.
